# Population Structure of Lenok (*Brachymystax savinovi*) in Lake Markakol and Preliminary Strategies for Species Conservation and Population Recovery

**DOI:** 10.3390/ani16162558

**Published:** 2026-08-16

**Authors:** Arailym Umirtayeva, Barakov Rinat, Kuanysh Isbekov, Saule Zh. Assylbekova, Aibek Kassymkhanov, Almat Suyubayev, Moldir Aubakirova, Angsar Satbek, Danil Kostyuchenko, Yerbol Sansyzbayev

**Affiliations:** 1 Department of Zoology, Histology and Cytology, Al-Farabi Kazakh National University, Almaty 050040, Kazakhstan; umirtayeva@fishrpc.kz (A.U.); satbek@fishrpc.kz (A.S.); 2 Fisheries Research and Production Center LLP, Almaty 050016, Kazakhstan; isbekov@fishrpc.kz (K.I.); assylbekova@fishrpc.kz (S.Z.A.); suyubaev@fishrpc.kz (A.S.); sansyzbayev@fishrpc.kz (Y.S.); 3 Altai Branch of the Fisheries Research and Production Center LLP, Ust-Kamenogorsk 070018, Kazakhstan; kasymhanov@fishrpc.kz (A.K.); kostyuchenko@fishrpc.kz (D.K.); 4 Balkhash Branch of the Fisheries Research and Production Center LLP, Balkhash 100300, Kazakhstan

**Keywords:** *Brachymystax savinovi*, population structure, conservation management, aquaculture, Lake Markakol

## Abstract

The Markakol lenok, *Brachymystax savinovi*, is an endemic salmonid inhabiting Lake Markakol and its tributaries in eastern Kazakhstan. Over the past century, both abundance and population structure of this taxon have been influenced by natural and anthropogenic factors, highlighting the need for updated assessments of its conservation status. This study evaluated the current population condition, habitat characteristics, and conservation prospects during 2020–2024. Results indicated that Lake Markakol maintained favourable hydrochemical and hydrological conditions during 2020–2024, with no evidence of substantial ecological degradation. The Markakol lenok population was characterised by the predominance of intermediate age classes, positive allometric growth, satisfactory condition factors, and a substantial proportion of mature individuals, indicating relatively stable population status and preserved reproductive potential. In addition, broodstock and replacement populations established under aquaculture conditions showed high survival and successful adaptation to different rearing technologies, confirming the feasibility of artificial propagation programmes for the conservation and sustainable management of the species. Wild brood fish from the Uidene River were successfully adapted to captive conditions, enabling establishment of broodstock and production of viable offspring under controlled conditions. These findings provide a scientific basis for conservation and restoration measures.

## Simple Summary

The Markakol lenok *(Brachymystax savinovi)* is a unique fish taxon occurring exclusively in Lake Markakol and its tributaries in eastern Kazakhstan. Over recent decades, its abundance has declined because of fishing pressure and environmental changes, highlighting the need for effective conservation measures based on long-term scientific evidence. This study presents the results of investigations conducted during 2020–2024 to evaluate the current population status, habitat conditions, and conservation prospects of this endemic salmonid. The results showed that the population is dominated by individuals of intermediate age classes, while large, older fish occur less frequently than in the past. Nevertheless, the species continues to exhibit positive allometric growth (b = 3.09), a high Fulton’s condition factor (FCF = 1.36 ± 0.15), and a substantial proportion of mature individuals, indicating preserved reproductive potential. Hydrochemical and hydrological conditions of Lake Markakol remained favourable throughout the study period, supporting the persistence of the species. The study also demonstrated that wild-caught lenok successfully adapted to aquaculture conditions, allowing the establishment of broodstock and replacement populations with high survival and the production of viable offspring under controlled conditions. These findings provide a scientific basis for the conservation and recovery of the Markakol lenok and demonstrate that integrating long-term population monitoring with ex situ conservation and artificial propagation represents an effective strategy for safeguarding this endemic freshwater salmonid.

## 1. Introduction

Species of the genus *Brachymystax* are among the most widely distributed cold-water salmonids in northern Asia and represent an important model for studies of phylogeography, evolutionary history, and adaptation to freshwater environments [1]. Recent genetic and genomic studies have revealed substantial population differentiation and cryptic diversity within the genus, emphasizing the importance of geographically isolated populations for understanding evolutionary processes and species conservation [2]. Furthermore, many lenok populations have experienced declines associated with habitat degradation, hydrological alteration, overfishing, and climate change, highlighting the need for continued monitoring and conservation-oriented research on endemic taxa such as *Brachymystax savinovi* [3]. 

The Markakol lenok (*Brachymystax savinovi*) is an endemic form of the Lake Markakol basin and represents a unique component of the regional ichthyofauna that was historically characterised by high abundance [4,5]. This taxon was originally described by Mitrofanov in 1959 based on its morphological distinctiveness and geographic isolation [4], although its taxonomic status remains a subject of ongoing discussion [6]. According to historical records, the total stock of lenok in the lake was estimated at approximately 1.5 thousand tonnes at the beginning of the twentieth century, while the maximum recorded commercial catch reached about 100 tonnes in 1986 [7,8]. The Markakol lenok inhabits Lake Markakol, which is located within the Markakol State Nature Reserve, a specially protected natural area [9].

Despite its protected status, the species remains socio-economically important to local communities and has historically contributed to local livelihoods [10].

Currently, lenok harvest is strictly regulated and permitted only under specific conservation requirements and for residents of designated settlements in accordance with the Law of the Republic of Kazakhstan «On Specially Protected Natural Areas» [11].

Annual catch limits for lenok constitute an important tool for the sustainable regulation of recreational and amateur fisheries in Lake Markakol. Nevertheless, the population of this species continues to experience anthropogenic pressures associated with fishing activities, alterations in the hydrological regime, and aquatic pollution [12,13,14]. According to the Markakol State Nature Reserve, incidents of illegal, unreported and unregulated (IUU) fishing targeting lenok and other fish species have been recorded in Lake Markakol [15]. These incidents highlight the continuing need for effective conservation, monitoring, and enforcement measures. The combined effects of anthropogenic and environmental pressures on salmonid populations have been widely documented worldwide and are considered major drivers of population decline and biodiversity loss in freshwater ecosystems, particularly in cold-water lake and river habitats occupied by salmonids [16,17,18]. 

The fish community of Lake Markakol, and particularly the endemic lenok as the dominant species in catches, plays an important role in supporting regional food security by serving as a source of food for local communities, which are predominantly rural. However, monitoring data are not always comprehensively assessed owing to the difficulties associated with field sampling, particularly within protected natural areas [19,20]. The aim of this study was to assess the current status of the Markakol lenok (*Brachymystax savinovi*) population and the hydrological and hydrochemical conditions of Lake Markakol, and to evaluate prospects for species conservation and population recovery using artificial propagation and aquaculture approaches.

In recent decades, aquaculture has developed considerably, with advances in production efficiency, environmental sustainability, and biosecurity [21]. In addition to its contribution to food production, aquaculture is increasingly recognised as an important tool for the conservation and recovery of depleted, rare, and endangered fish populations [22,23].

This study was based on the hypothesis that the current status of the Markakol lenok (*Brachymystax savinovi*) population is determined by a combination of environmental factors in Lake Markakol and anthropogenic pressures, including recreational fishing. We hypothesised that a comprehensive assessment of the species’ biological characteristics and the lake’s hydrological and hydrochemical conditions would provide a robust scientific basis for developing effective conservation and management measures. The findings of this study contribute to the improvement of conservation strategies, artificial propagation programmes, and biodiversity management within the Lake Markakol ecosystem.

## 2. Materials and Methods

### 2.1. Characteristics of Lake Markakol

Lake Markakol is a high-altitude cold-water lake (1449.3 m a.s.l.) with a maximum depth of 24–27 m and numerous inflowing tributaries that maintain its hydrological regime [24]. The lake remains ice-covered for approximately six months each year due to the severe climatic conditions of the Markakol Basin, one of the coldest regions of Kazakhstan [25]. These hydrological and thermal conditions are characteristic of habitats occupied by cold-water salmonids and provide an important environmental context for assessing the status of the endemic Markakol lenok.

### 2.2. Research Area and Data Collection

**Figure 1:**
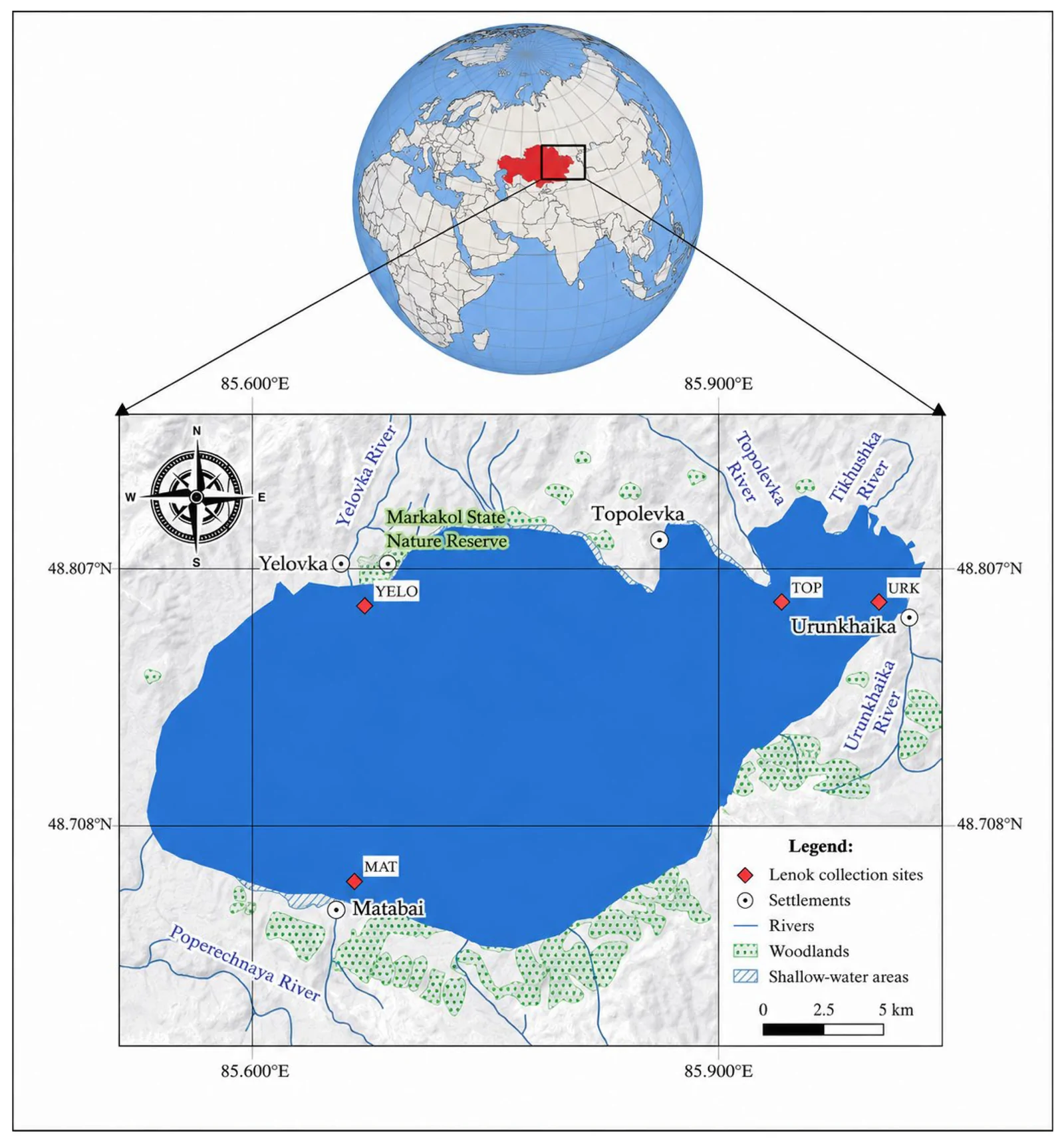
Map of sampling sites and catches of Asian lenok (*Brachymystax lenok*) in Lake Markakol in 2020–2024. Abbreviations: URK—Urunkhaika, TOP—Topolevka, YELO—Yelovka, MAT—Matabay.

Samples of Markakol lenok used to assess biological and population characteristics were collected from Lake Markakol during 2020–2024 using multi-mesh gill nets. Gill nets were deployed in the littoral and sublittoral zones of Lake Markakol at depths ranging from 2 to 10 m. Nets were set in the evening and left overnight for approximately 12 h. Fish were collected and processed during the morning hours following net retrieval. Field surveys were conducted at four permanent sampling stations–Urunkhaika, Topolevka, Yelovka and Matabay (Figure 1) during three seasonal periods: spring, summer, and autumn.

**Figure 2:**
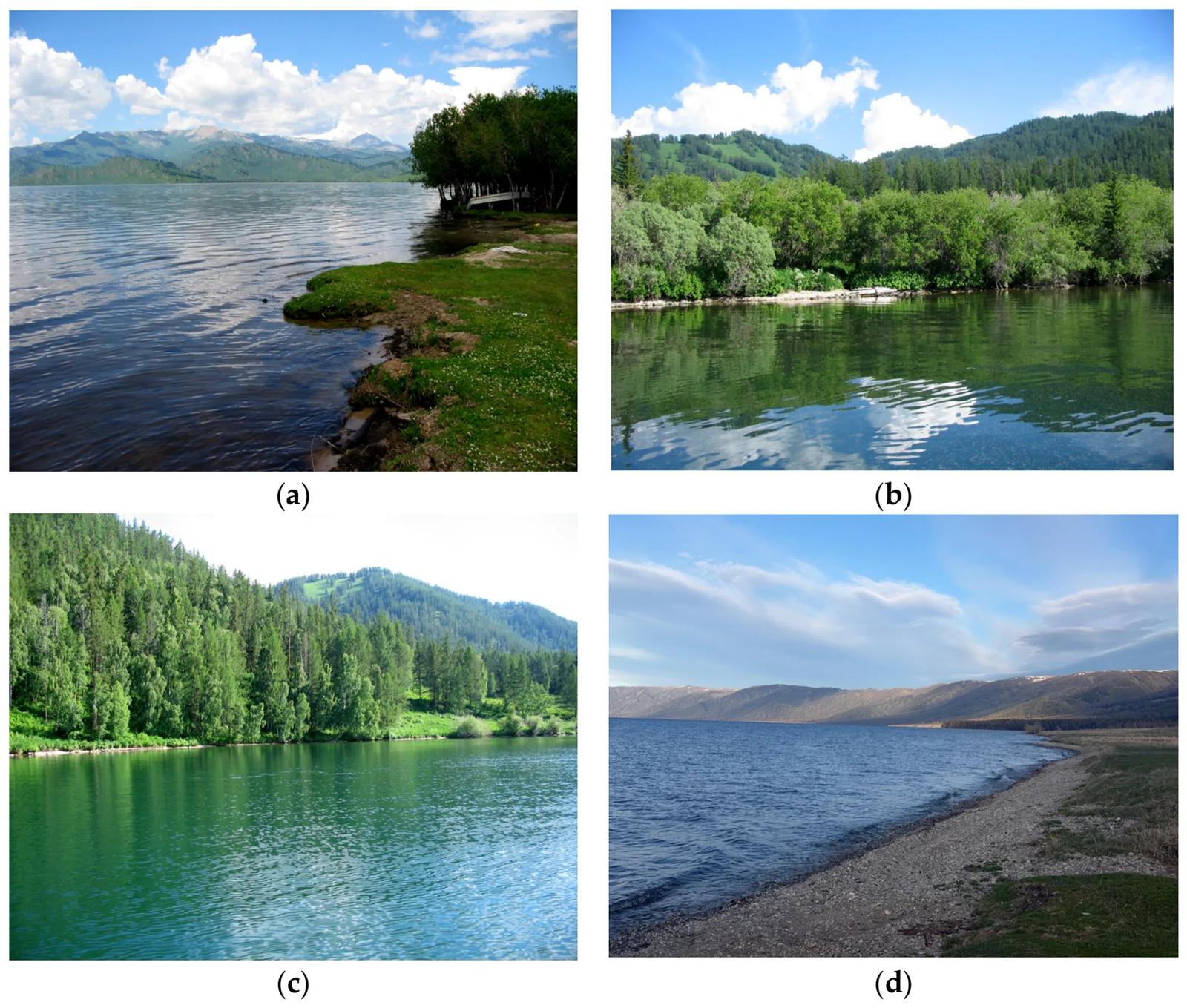
Selected areas of the Lake Markakol shoreline and aquatic habitats in the vicinity of the lenok sampling stations: (**a**)—area near the mouth of the Urunkhaika River; (**b**)—area of the Topolevka station; (**c**)—access route to the Yelovka station; (**d**)—coastal area near the Matabay station.

The lenok sampling stations encompassed areas adjacent to the mouths of the principal tributaries of Lake Markakol, including the Urunkhaika, Topolevka, Elovka, Matabai, and Poperechnaya rivers. Representative sections of the lake located in the vicinity of the lenok sampling stations are shown in Figure 2.

Following capture, fish were sorted by species and size class. The coordinates of the lenok sampling stations are presented in Table 1. A total of 1993 lenok specimens were collected during the study period from 2020 to 2024. 

The main habitat characteristics of the lenok sampling stations in Lake Markakol are summarized in Table 1. The selected stations were located in different sectors of the lake and represented a range of environmental conditions, including variation in depth, bottom substrate composition, shoreline configuration, and the influence of tributary inflows. 

Hydrochemical data for Lake Markakol were obtained from annual monitoring reports of LLP «Fisheries Research and Production Centre» (FRPC) for the period 2020–2024 [26]. The hydrochemical status of the lake was assessed using the following water quality parameters: pH, free carbon dioxide concentration (CO_2_), dissolved oxygen concentration (DO), dissolved oxygen saturation (DO sat), ammonium nitrogen (NH_4_^+^), nitrite (NO_2_^−^), nitrate (NO_3_^−^), phosphate (PO_4_^3−^), permanganate index (PI), and total dissolved solids (TDS).

Primary hydrological data, including records of water-level fluctuations in Lake Markakol for the period 2020–2024, were obtained from the East Kazakhstan and Abai Regional Branch of the Republican State Enterprise «Kazhydromet» [27]. The analysis of water-level dynamics was based on conventional gauge-level measurements expressed in metres (m).

### 2.3. Catch per Unit Effort (CPUE)

Catch per unit effort (CPUE) was used as an index of relative abundance and was calculated as the catch biomass (kg) per standardized gill-net set. Sampling effort was standardized by using the same type of fishing gear, mesh-size composition, and approximately identical soaking time (12 h overnight) throughout the study period [28]. Thus, CPUE was expressed as kg·net − 1·night − 1 and was calculated as:

CPUE = C/E,

where C is the total catch biomass (kg), and E is the fishing effort (number of net-nights). The approach follows standard fisheries methodology, where CPUE is calculated as catch relative to a standardized unit of fishing effort and used as an indicator of relative stock abundance.

### 2.4. Lenok Capture for Ex Situ Conservation

**Figure 3:**
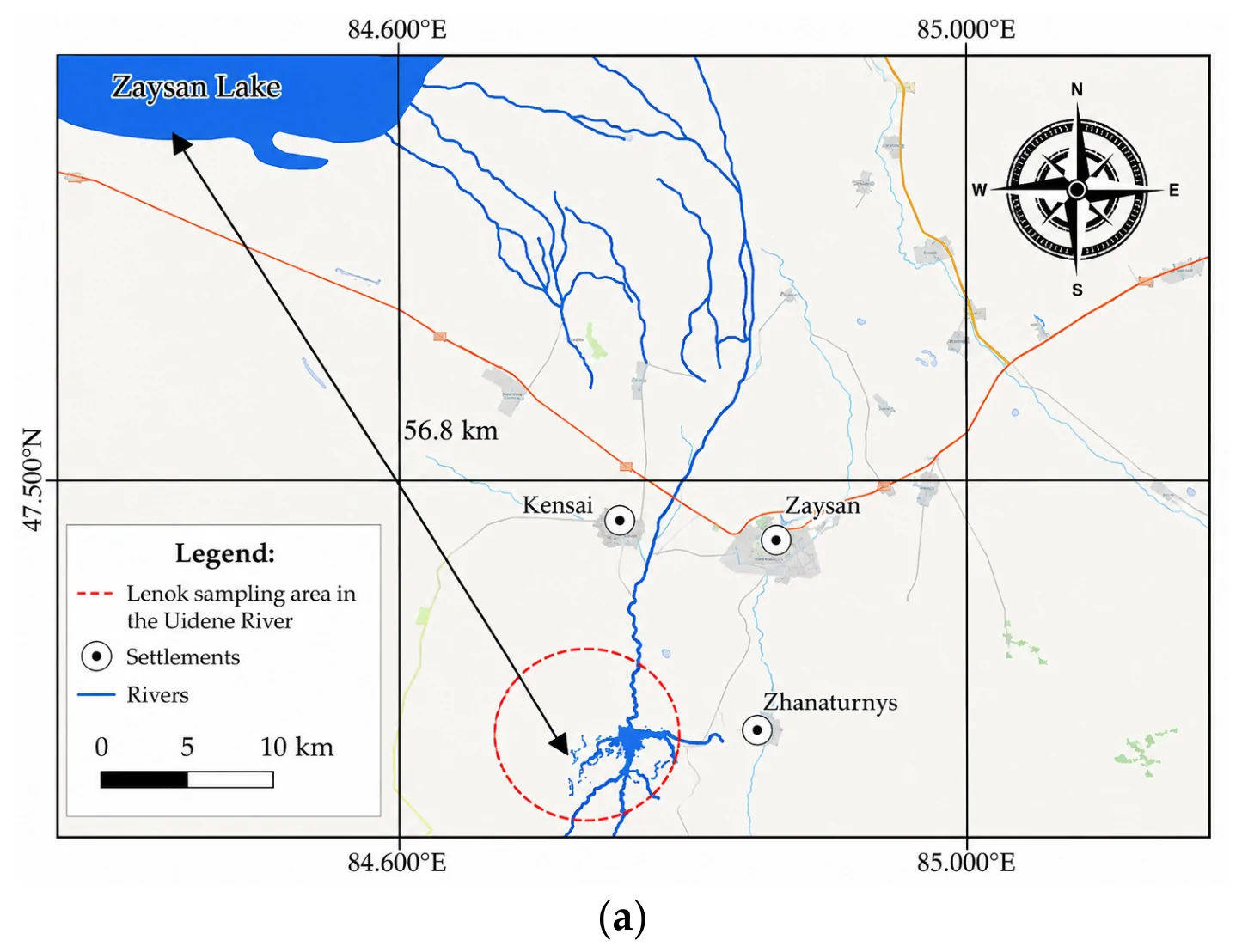

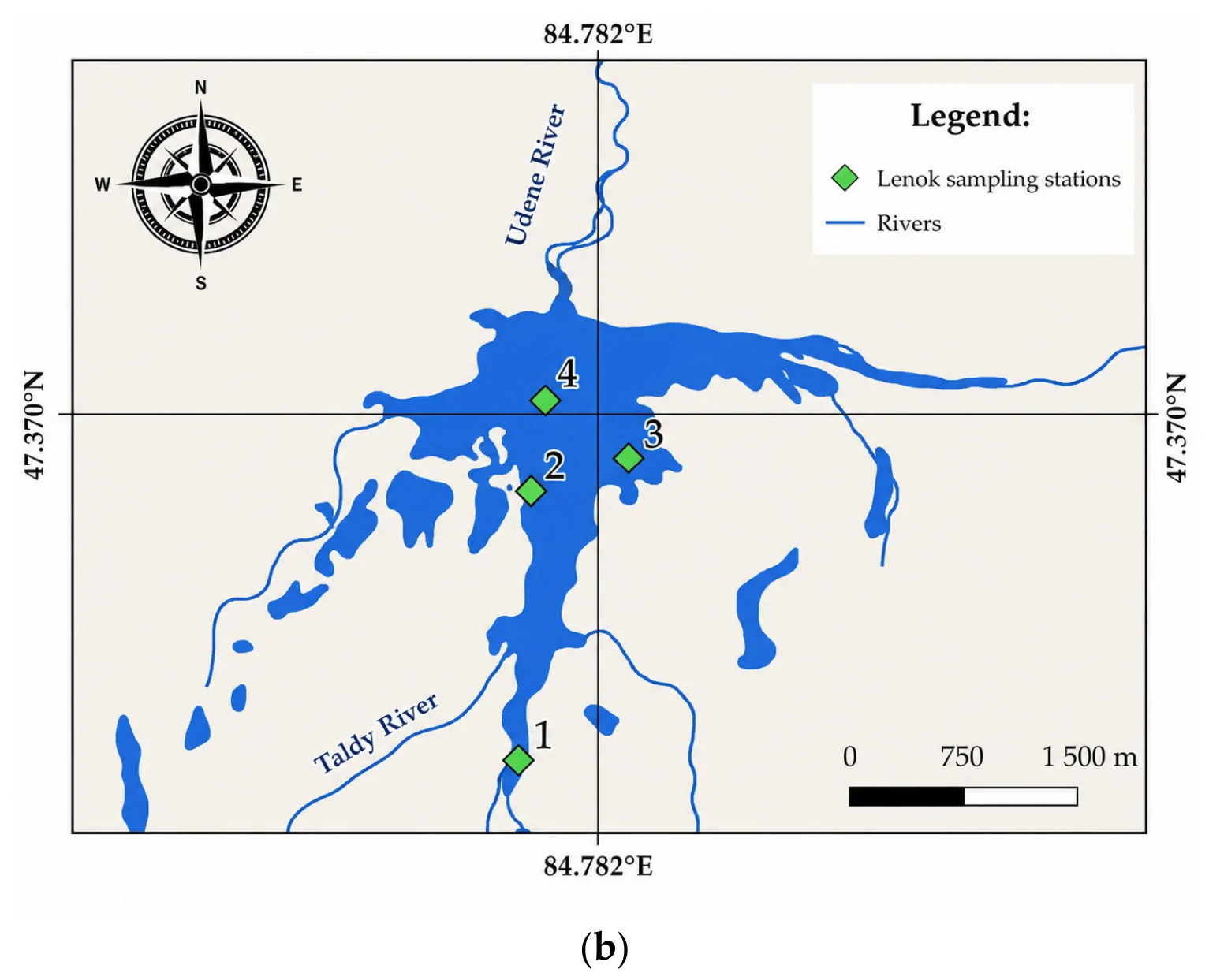
Schematic map of lenok sampling in the Uidene Reservoir: (**a**) location of the Uidene Reservoir relative to Lake Zaysan; (**b**) lenok sampling stations.

Lenok intended for the establishment of a replacement and broodstock population were collected from the Uidene River Reservoir in 2022 at four sampling stations during the summer and autumn seasons (Figure 3). The reservoir has a storage capacity of 75 million m^3^ and plays an important role in regulating the hydrological regime of the study area [29].

Lenok is widely distributed throughout the Uidene River Reservoir and, during the summer period, tends to concentrate in the central part of the waterbody. Juvenile lenok (age 0+) originating from Lake Markakol were repeatedly released into the Uidene River Reservoir by local residents of the city of Zaisan in the early 1970s [25]. Both populations belong to the same species and share a common origin. However, long-term exposure to different environmental conditions has resulted in differences in plastic and meristic traits, as well as in the timing of sexual maturation. The Uidene Reservoir population is characterized by relatively larger head and eye proportions, lighter body coloration, and larger but less numerous spots concentrated mainly on the dorsal region. These differences are most likely associated with contrasting abiotic conditions and represent intraspecific phenotypic variation rather than genetic divergence. Consequently, individuals from this reservoir were selected for the establishment of a broodstock population [30]. A total of 105 lenok specimens were collected in 2022. Subsequent domestication was carried out under aquaculture conditions at OstFish LLP, operating a recirculating aquaculture system (RAS), and at Grand-FISH LLP, which operates a flow-through tank-based aquaculture facility.

#### Characteristics of Lenok Rearing Conditions

Domestication of Markakol lenok under recirculating aquaculture system (RAS) conditions was carried out at OstFish LLP from June to December 2022. The initial body weight of fish in this group ranged from 503.0 to 1122.0 g. Water temperature at the inflow and within the rearing tanks remained stable throughout the study period, varying between 12.4 and 16.7 °C. Dissolved oxygen concentrations at the water inflow ranged from 8.6 to 10.2 mg/L, corresponding to favourable conditions for the maintenance and growth of salmonid fishes.

The replacement and broodstock population of Markakol lenok was fed commercially formulated complete diets produced by the Danish company Aller Aqua (manufactured in Poland). The principal production feed used during the rearing period was Aller Bronze with a pellet size of 2.0 mm. This diet is specifically formulated for salmonid species and was therefore selected for the cultivation of Markakol lenok [31].

**Figure 4:**
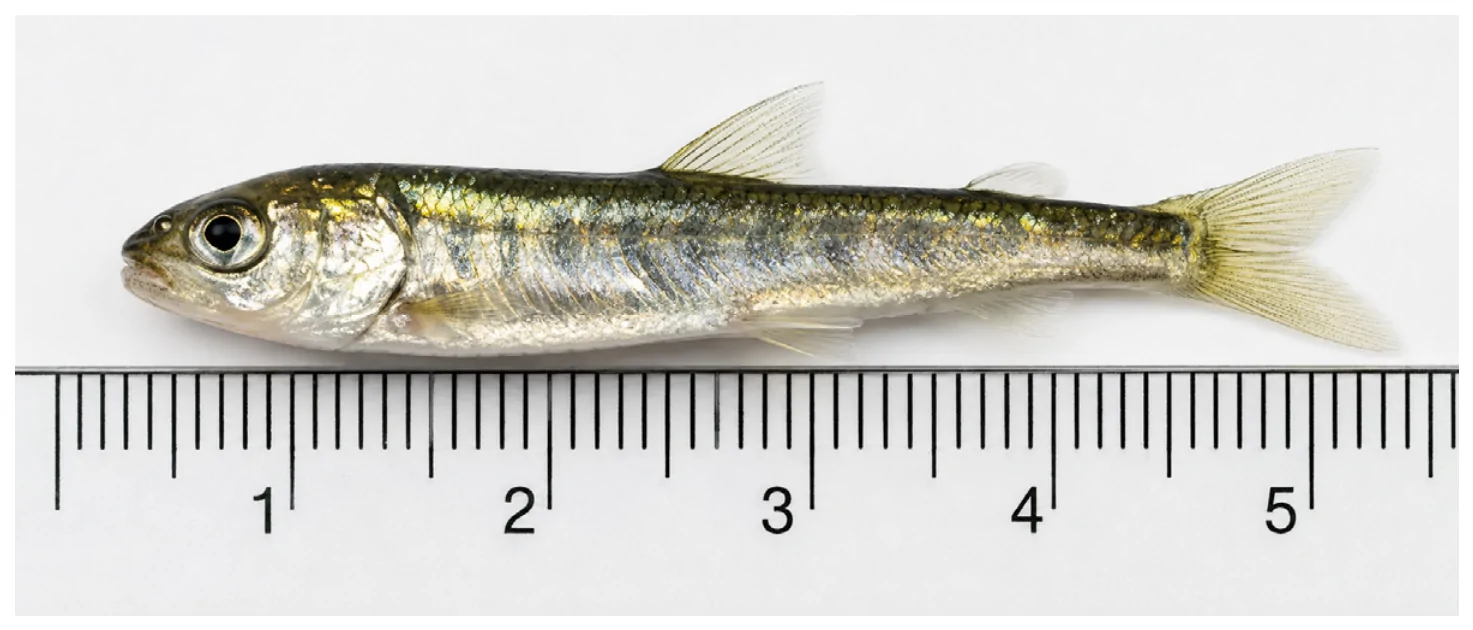
Representative specimen *Brachymystax savinovi* from the second experimental group reared at Grand-FISH LLP.

The second group of lenok, with an initial body weight ranging from 138.0 to 417.0 g, was maintained in flow-through rearing tanks at Grand-FISH LLP (Figure 4). The total rearing period for this group was 120 days. Water temperature in the rearing tanks remained relatively stable throughout the study period, varying between 8.2 and 10.4 °C, which was slightly higher than the temperatures typically observed in the natural environment during winter. Dissolved oxygen concentrations met the requirements for salmonid culture and ranged from 7.5 to 11.6 mg/L. Fish were fed Aller Silver feed with pellet sizes ranging from 3.0 to 6.0 mm, depending on the body size and weight of the lenok [32].

### 2.5. Biological Analysis

Biological analyses of captured lenok specimens were conducted in accordance with the widely accepted methodological guidelines of Pravdin [33]. Morphometric measurements were performed using non-lethal methods. The principal biological characteristics recorded included total length (TL), body length from the tip of the snout to the base of the caudal fin (l), and total body weight (Q). Fish age was determined by scale analysis following standard procedures for salmonid fishes [34]. Age was determined for all lenok specimens collected during the study period (2020–2024), comprising a total sample of 1993 individuals. For each fish, 5–10 scales were removed, cleaned, and examined under magnification. Age estimates were based on the interpretation of annual growth rings (annuli), and the most clearly readable scales were used for final age determination. To minimise reading errors, age estimates were independently verified through repeated readings.

#### 2.5.1. Aggregation Index

To assess the spatial distribution and degree of aggregation of lenok in fishery catches, the Aggregation Index (AI) was employed. This index is based on the ratio of the variance to the mean abundance and was calculated according to the following equation [35]:

$$\mathrm{AI}=S^{2}/\overset{-}{X}$$

where AI is the Aggregation Index; S^2^ is the variance of the number of individuals per catch; and $\overset{-}{X}$ is the mean number of individuals per catch.

Values of AI > 1 indicate an aggregated distribution pattern; values close to AI ≈ 1 correspond to a random distribution, whereas values of AI < 1 indicate a more uniform distribution of fish among catches.

#### 2.5.2. Length–Weight Relationships

The length–weight ratio (TL/TW) of lenok was calculated using the Bagenal and Tesch formula [36]:

TW = aTL^b^

where

TW = Total Weight (g)

TL = Total Length (cm)

a = Intercept of the regression line

b = Slope of the regression line

Length–weight relationships were analysed using total length and body weight measurements collected from 1993 lenok specimens. The individual body length and body weight measurements of 1993 specimens collected during 2020–2024 were classified according to the determined age of each specimen. For each age group within each study year, the mean body length and body weight were calculated. The resulting mean body length and body weight values corresponding to the respective age groups in each study year were subsequently used to construct the length–weight relationship. The coefficient of determination (R^2^) was calculated from the same aggregated mean values of body length and body weight used to fit the length–weight model and was used to assess the goodness of fit of the relationship.

Following the length–weight relationship analysis, the parameters of the length–weight relationship model (TW = aTL^b^) were estimated to characterise the growth pattern of Markakol lenok and to determine whether growth was isometric or allometric, following the approach of Le Cren [37].

#### 2.5.3. Condition Factor (Fulton’s Factor)

The body condition of lenok was assessed using Fulton’s condition factor according to the method described in [38]. The coefficient was calculated using the following formula:

$$\mathrm{FCF}\;=\;\frac{TW}{{TL}^{3}}\times 100$$

where TW is the total weight in grams, and TL is the total length in centimeters. 

Fulton’s condition factor was calculated for all individuals included in the sample for each study year (2020–2024). The analysis was performed using pooled annual samples, without subdivision by sex, age group, or season. Fulton’s condition factor (FCF) was calculated for all captured lenok specimens collected during 2020–2024 (N = 1993). Mean annual FCF values were subsequently calculated and compared among study years to evaluate temporal variation in the physiological condition of the population.

#### 2.5.4. Sex Determination and Gonadal Maturity Assessment

Sex and gonadal maturity were assessed through macroscopic examination of the gonads after dissection, following the methodology described by Pravdin [33]. The analysis included 354 specimens (N = 354) collected during the reproductive season (April–May) from 2020 to 2024. Sex was determined based on the external morphology and development of the gonads. Gonadal maturity was evaluated according to the stage of gonad development, with individuals at stages I–II classified as immature and those at stages III–VI considered sexually mature.

#### 2.5.5. Estimation of Length at Sexual Maturity (L25, L50, L75)

Maturity status was determined from gonadal development stages, with individuals classified as mature (stages III–VI) or immature (stages I–II). A total of 354 specimens (N = 354) collected during the reproductive season (April–May) between 2020 and 2024 were used for the analysis. The proportions of mature individuals were modelled as a function of total length using logistic regression, and the lengths at which 25%, 50%, and 75% of individuals attained sexual maturity (L_25_, L_50_, and L_75_) were estimated from the fitted model.

To evaluate size-related maturation parameters in lenok, a logistic model describing the relationship between the probability of maturation and fish body length was applied [39]. The observed probability of maturation (Observed P) was calculated using the following equation:

Observed P = nm/N

where Observed P is the observed probability of maturation; *n*_m_ is the number of mature individuals within a given length class; and *N* is the total number of individuals examined in that length class.

The modelled probability of maturation (Model P) was estimated using a logistic function:

Model P = 1/(1 + e − (a + bL))

where *L* is the body length of the fish; *a* and *b* are the coefficients of the logistic regression model; and e is the base of the natural logarithm.

Based on the fitted logistic model, the parameters L25, L50, and L75 were calculated, representing the body lengths at which the probability of maturation reaches 25%, 50%, and 75%, respectively:

Lp = [ln(P/(1 − P)) − a]/b

where *L*_p_ is the body length at a given probability of maturation (*P*); *P* is the probability of maturation (0.25, 0.50, or 0.75); ln denotes the natural logarithm; and *a* and *b* are the coefficients of the logistic model.

### 2.6. Statistical Data Analysis

Statistical analyses were performed using IBM SPSS Statistics software (2022 version) [40]. Descriptive statistical parameters were calculated, including the mean (Mean), coefficient of variation (CV), and standard deviation (±SD).

Statistical analysis and visualisation of hydrochemical data were performed using PAST software, version 4.03 [41]. Principal Component Analysis (PCA) was applied to identify the main patterns of variation in the hydrochemical characteristics of Lake Markakol and to determine the variables contributing most strongly to interannual differences in water quality. The analysis included the principal hydrochemical parameters measured during the study period, including water temperature, dissolved oxygen, pH, mineralisation, and major ion concentrations. The interpretation of PCA results was based on the distribution of samples in the ordination space and the loadings of environmental variables on the principal components.

Linear regression analysis was applied to identify temporal trends in selected hydrological and hydrochemical variables of Lake Markakol. The coefficient of variation (CV) was calculated to assess the variability of river discharge. Relationships among hydrological and hydrochemical parameters, lenok catch abundance, and fishing effort were evaluated using Pearson’s correlation coefficient (*r*) and Spearman’s rank correlation coefficient (*ρ*) [42,43].

## 3. Results

### 3.1. Hydrochemical Parameters of Water

The hydrochemical characteristics of Lake Markakol are presented in Table 2. The lake water was characterised by neutral to slightly alkaline conditions, with pH values ranging from 7.50 ± 0.07 (2020 and 2023) to 8.40 ± 0.08 (2024). Concentrations of free carbon dioxide (CO_2_) remained stable throughout the study period at approximately 0.10 mg/L. These values were consistent with the hydrochemical characteristics recorded for Lake Markakol throughout the study period. Dissolved oxygen concentration (DO) and dissolved oxygen saturation (DO sat) remained high across all sampling years, indicating stable oxygen conditions in the lake.

Concentrations of nutrient compounds (NH_4_^+^, NO_2_^−^, NO_3_^−^, and PO_4_^3−^) did not exceed the permissible limits established for fishery water bodies [44]. Nevertheless, ammonium (NH_4_^+^) and nitrate (NO_3_^−^) concentrations were higher than those of the other measured nutrient components. Ammonium concentrations ranged from <0.20 mg/L in 2024 to 0.54 ± 0.08 mg/L in 2020, whereas nitrate concentrations varied from <0.01 mg/L in 2020 to 0.66 ± 0.05 mg/L in 2021.

Permanganate index (PI) values ranged from 1.54 ± 0.12 mg O/L in 2023 to 1.93 ± 0.18 mg O/L in 2022. The highest level of total dissolved solids (TDS) was also recorded in 2022, reaching 63.00 ± 2.80 mg/L. The elevated PI values recorded in 2022 may reflect a temporary increase in the input of organic matter into the aquatic ecosystem. Overall, the limited interannual variability of PI and TDS suggests a relatively stable hydrochemical regime in Lake Markakol.

Linear regression analysis was applied to assess potential trends in the hydrochemical characteristics of Lake Markakol. The results of the linear regression analysis for hydrochemical parameters measured during 2020–2024 are presented in Table 3.

For all hydrochemical variables examined, the identified trends should be regarded as preliminary owing to the limited sample size and the lack of statistical significance (*p* > 0.05). Nevertheless, several parameters exhibited temporal patterns that warrant further investigation. In particular, ammonium (NH_4_^+^) showed a tendency towards increasing concentrations over time (slope *B* = 0.81, *p* > 0.05), while nitrite concentrations displayed a weak positive trend (slope *B* = 0.72, *p* > 0.05). Dissolved oxygen saturation also demonstrated a moderate increase during the study period (slope *B* = 0.29, *p* > 0.05). In contrast, pH values exhibited a decreasing trend over time (slope *B* = −1.60, *p* > 0.05). No pronounced trend was observed for the permanganate index, which remained relatively stable throughout the study period (slope *B* = −1.45, *p* > 0.05).

**Figure 5:**
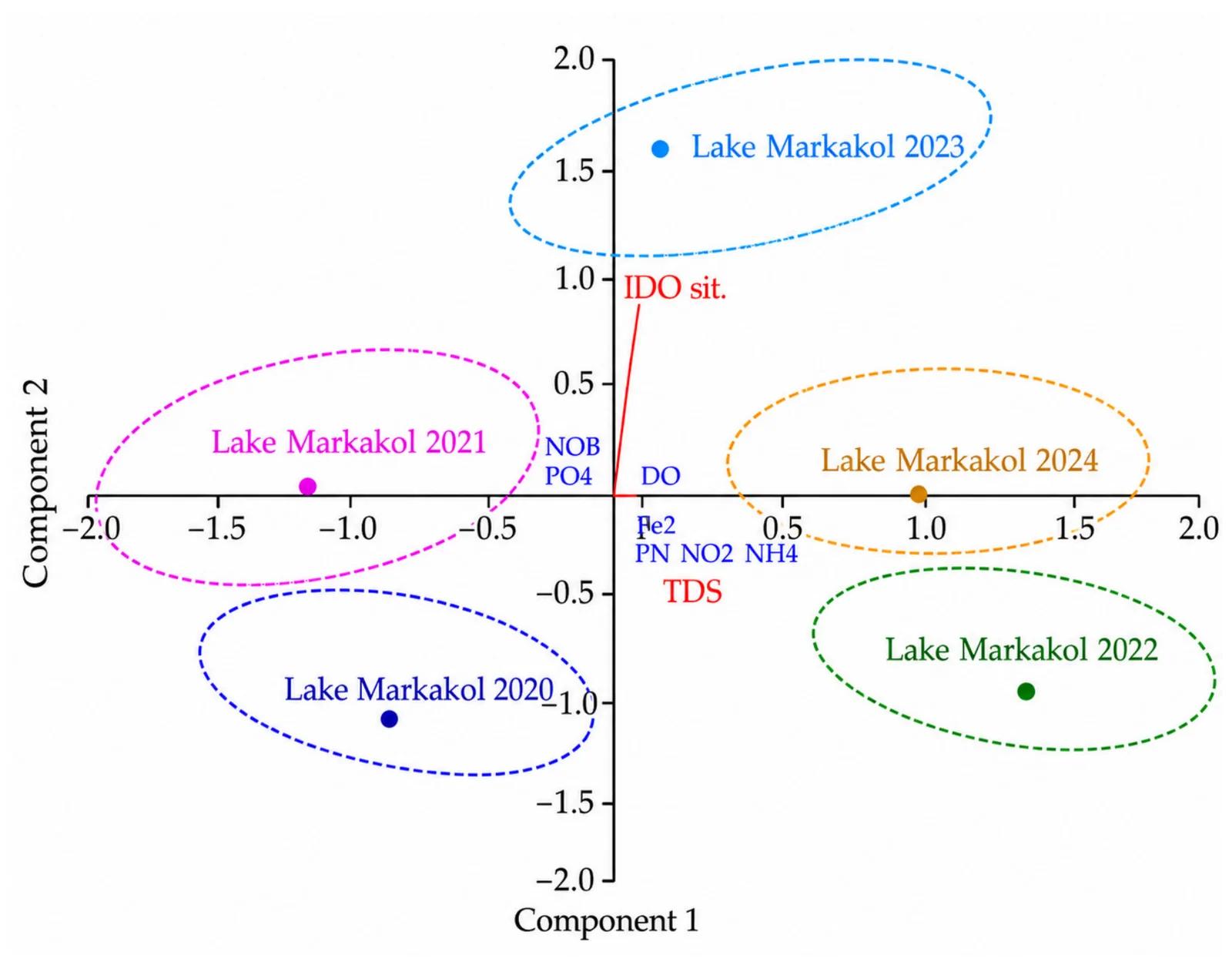
Interannual variability of hydrochemical parameters in Lake Markakol water during 2020–2024. Red color indicates the direction of the most pronounced changes in hydrochemical parameters. Abbreviations: DO—dissolved oxygen; DO sat—oxygen saturation; CO_2_—carbon dioxide; NH_4_—ammonium nitrogen; NO_2_—nitrite nitrogen; NO_3_—nitrate nitrogen; PO_4_—phosphates; PI—permanganate index; TDS—total dissolved solids.

As shown in Figure 5, samples collected during 2020–2021 form a distinct cluster separate from those obtained in 2022–2024, reflecting differences in hydrochemical conditions associated with lower concentrations of NH_4_^+^ and NO_2_^−^, as well as lower TDS values during the earlier period. In contrast, samples from 2023 constitute an independent cluster characterised by elevated dissolved oxygen concentrations (DO). The 2024 samples show a shift towards the 2023 cluster, suggesting a greater similarity in hydrochemical conditions between these two years.

Overall, the interannual dynamics of the hydrochemical characteristics of Lake Markakol during 2020–2024 indicate a degree of heterogeneity across the study period. Elevated dissolved oxygen saturation was observed in 2022, whereas the years 2023–2024 were characterised by a more stable hydrochemical regime and greater similarity in the values of most measured parameters. No substantial deterioration in water quality was detected during the study period, and the observed fluctuations are considered to reflect natural interannual variability in the hydrochemical conditions of the lake.

### 3.2. Hydrological Regime

**Figure 6:**
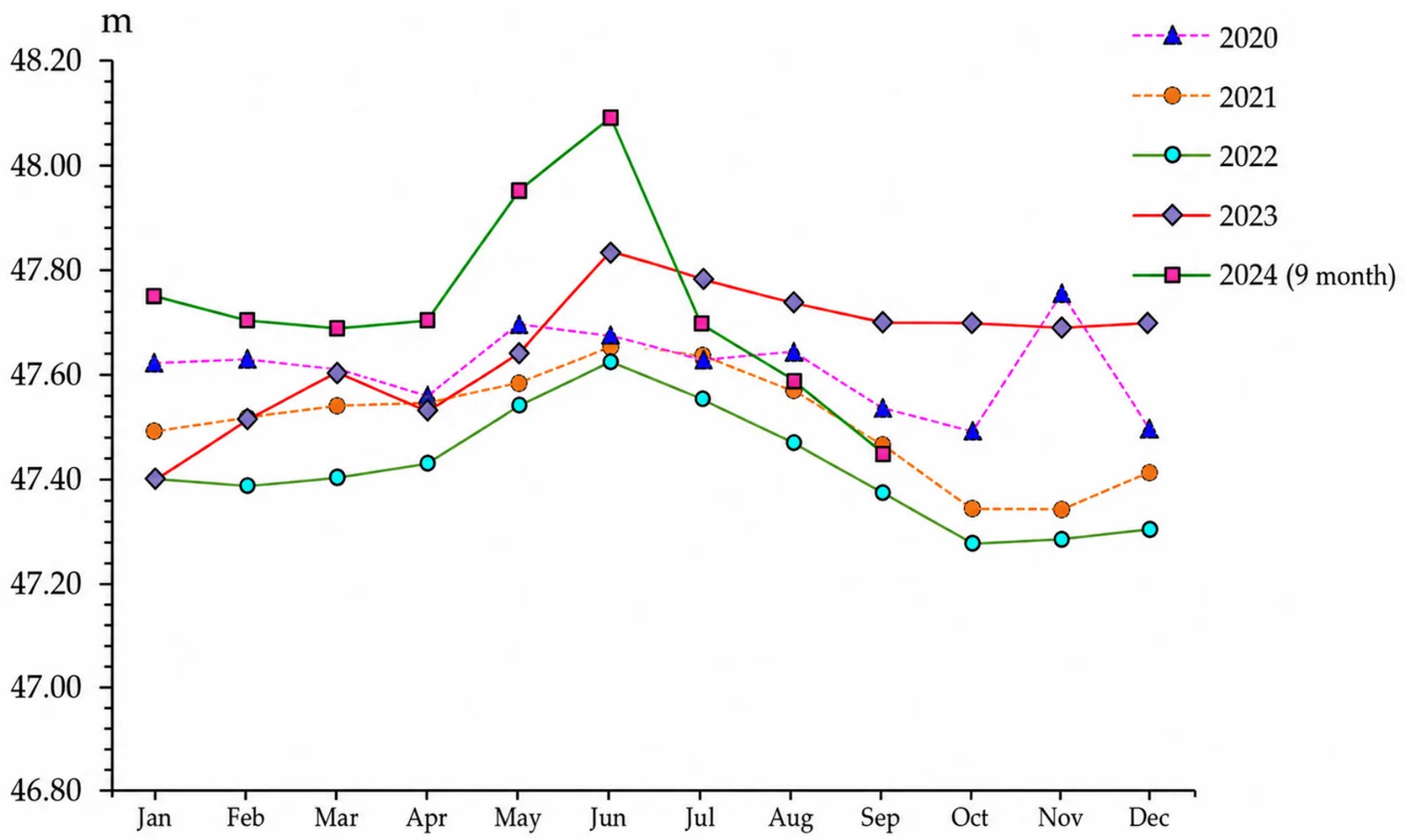
Dynamics of the water-level regime of Lake Markakol during 2020–2024. Seasonal and interannual fluctuations in Lake water level are presented based on hydrological monitoring data collected throughout the study period.

Seasonal variation in the hydrological regime of Lake Markakol is characterised by a rise in water level during the spring–summer period (April–June), followed by a gradual decline from mid-summer until the end of the year (Figure 6). The highest water level was recorded in June 2024, reaching 48.08 m, whereas the lowest levels were observed in 2022.

The observed seasonal pattern is primarily driven by increased inflow associated with spring snowmelt and precipitation, while the subsequent decline in water level reflects a reduction in discharge from the inflowing tributaries. The low water levels recorded in 2022 were likely associated with unfavourable hydrometeorological conditions, including reduced snowpack accumulation and lower precipitation within the lake basin. In contrast, 2024 was characterised by comparatively higher water levels, with a mean value of 47.74 ± 0.18 m, exceeding those recorded in the other years examined.

The water-level regime of Lake Markakol exhibited relatively low variability within individual years, as indicated by coefficients of variation that did not exceed 0.25% (Table 4). However, analysis of interannual dynamics revealed no statistically significant trends in water-level change for most of the study period (*p* > 0.05). A significant positive trend was detected only in 2023, indicating an increase in water level over time (*B* = 0.67; *p* <0.05).

Overall, the water-level regime of Lake Markakol during the study period was characterised by relatively stable intra-annual fluctuations and the absence of pronounced interannual changes. 

### 3.3. Assessment of Lenok Catches

**Figure 7:**
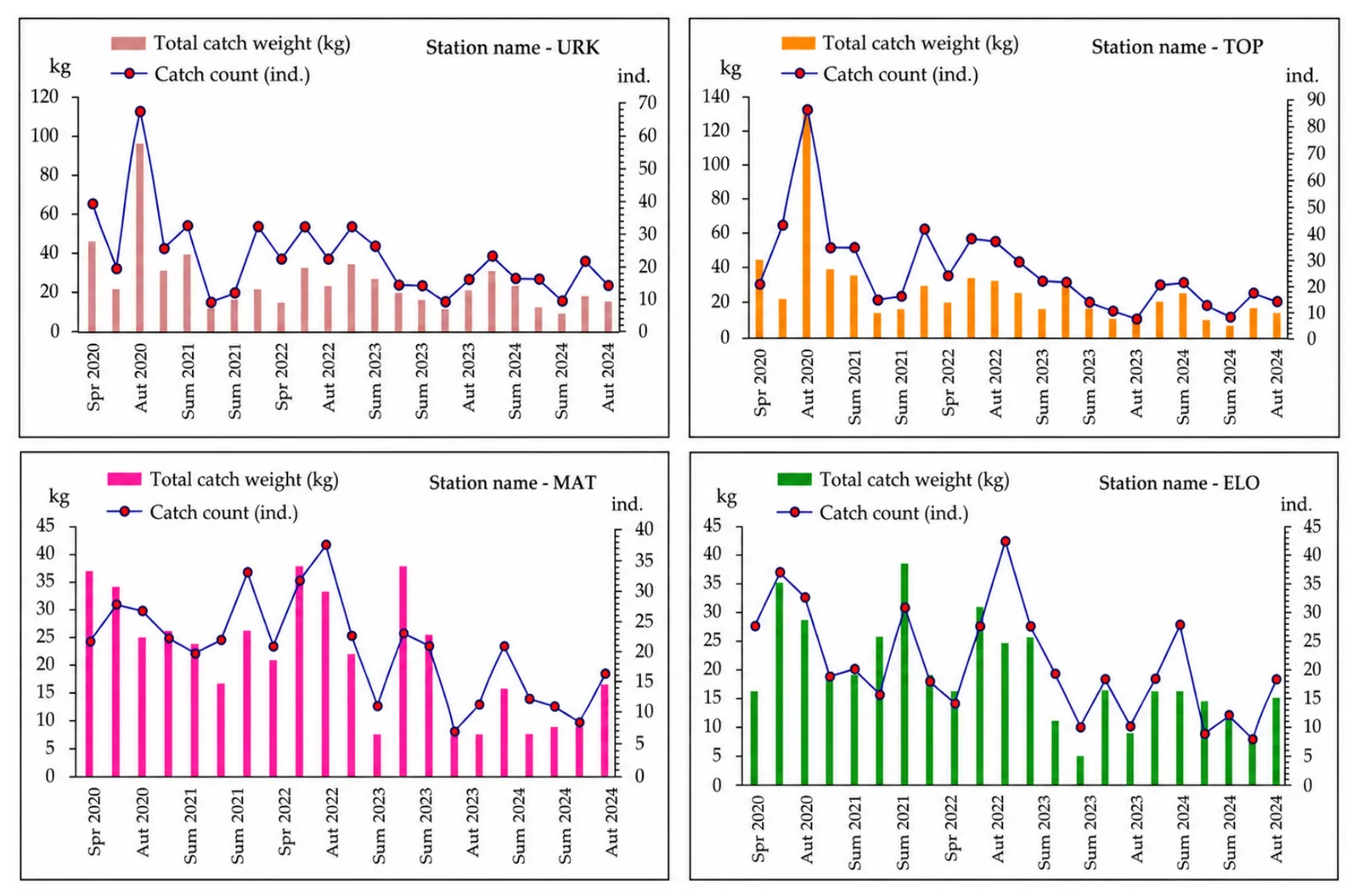
Spatial and seasonal variation in lenok catches among sampling stations in Lake Markakol during 2020–2024, including changes in abundance and biomass. Station abbreviations: URK, Urunkhaika; TOP, Topolevka; MAT, Matabai; ELO, Elovka.

The distribution of lenok in Lake Markakol exhibited pronounced interannual and seasonal variability (Figure 7). For each year, only the seasons in which sampling was conducted are presented in the figure. Sampling was conducted during several field expeditions between 2020 and 2024. The number of gill-net samples collected at each station varied among seasons because sampling intensity depended on weather conditions, field logistics, and fish availability. Consequently, in some seasons, two or more independent gill-net samples were collected at the same station. These samples are presented separately in Figure 7 to better reflect seasonal variation in the size structure of the examined fish. The observed differences were associated with the spatial location of sampling stations and the heterogeneity of abiotic conditions across different areas of the lake. The highest abundance of lenok was recorded during the autumn of 2020 at the Urunkhaika (URK) and Topolevka (TOP) stations, where 66 and 84 individuals were captured, respectively. In subsequent years, as well as during the spring and summer sampling periods of 2020–2024, lenok abundance at these stations was considerably lower.

At the Matabai (MAT) and Elovka (ELO) stations, a general increase in lenok abundance and total catch biomass was observed over much of the study period. However, both indicators declined at these stations during 2023–2024. In the summer season, lenok abundance at the Matabai and Elovka stations decreased to 10 and 7 individuals, respectively, suggesting a reduction in local aggregations of the species compared with previous sampling periods.

Correlation analysis revealed strong positive relationships between lenok abundance and total catch biomass at all sampling stations (*r* = 0.77–0.93; *p* < 0.01) (Table 5). These results indicate that increases in the number of captured individuals were accompanied by proportional increases in total catch biomass.

The strongest relationship was observed at the Urunkhaika station (URK; *r* = 0.93), followed by Topolevka (TOP; *r* = 0.90), Matabai (MAT; *r* = 0.83), and Elovka (ELO; *r* = 0.77). Overall, the findings suggest a relatively stable size–weight structure of lenok catches across different areas of Lake Markakol.

In addition to the relative stability of the population’s size–weight structure, the spatial distribution of lenok in Lake Markakol was characterised by an aggregated distribution pattern (Table 6). During certain seasons, fish were concentrated in specific areas of the lake, resulting in local increases in abundance and biomass. The formation of these aggregations is likely associated with seasonal aspects of the species’ biology, including feeding activity, reproductive behaviour, and preferences for particular environmental conditions.

**Figure 8:**
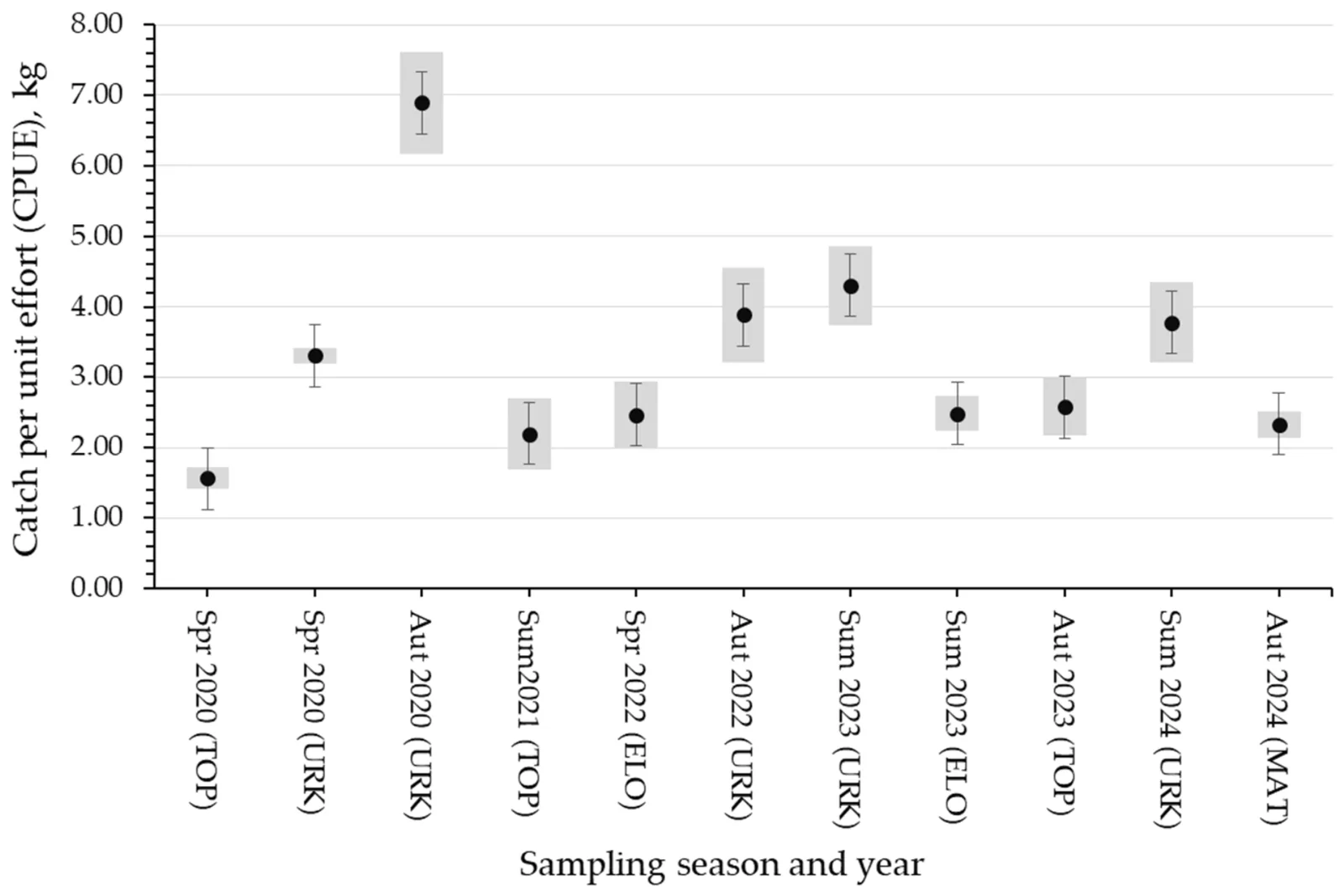
Distribution of lenok catch per unit effort (CPUE) across seasons and years during 2020–2024. Station abbreviations: URK, Urunkhaika; TOP, Topolevka; MAT, Matabai; ELO, Elovka.

The highest Aggregation Index was recorded in spring 2020 (AI = 5.21 ± 2.22), likely reflecting spawning-related movements of lenok and the formation of local aggregations in reproductive areas. Elevated aggregation values were also observed during the summer of 2021 and 2023 (AI = 3.84 ± 2.37 and 3.14 ± 2.27, respectively). These findings suggest a persistent tendency towards spatial concentration of fish in specific areas of the lake, which may be associated with feeding behaviour, seasonal movements, and the selection of favourable habitats. The distribution of lenok catch per unit effort (CPUE) by season and year during 2020–2024 is presented in Figure 8. Repeated seasonal labels correspond to different sampling stations surveyed during the same season and do not represent replicate measurements at a single station.

The highest mean CPUE was recorded at Urunkhaika in autumn 2020 (6.89), whereas the lowest was observed at Topolevka in spring 2020 (1.56). At Urunkhaika, mean CPUE values remained comparatively high throughout the study period, ranging from 3.31 to 6.89. In contrast, Topolevka generally showed lower CPUE values, ranging from 1.56 to 2.58. At Elovka, CPUE was relatively consistent between spring 2022 and summer 2023 (2.47 and 2.49, respectively), while the mean CPUE at Matabai in autumn 2024 was 2.33. The spatial and seasonal variation in CPUE suggests that the distribution of *Brachymystax savinovi* within Lake Markakol is heterogeneous, with Urunkhaika representing an area of relatively higher occurrence during the study period.

Overall, the dynamics of CPUE, together with the Aggregation Index (AI), indicate an aggregated distribution pattern of lenok within Lake Markakol and demonstrate that the degree of spatial concentration varies among seasons and years.

### 3.4. Effects of Water Level Fluctuations on Catch Indicators and Hydrochemical Parameters

No positive correlations were detected between water-level fluctuations and lenok catches, expressed either as CPUE or as the number of individuals recorded at the sampling stations (Table 7). This may be explained by the relatively stable water-level regime of Lake Markakol, while the increase in water levels observed in recent years may have contributed favourably to the reproductive success of lenok. In contrast, water level exhibited negative correlations with several other variables examined in the study.

For example, a strong inverse relationship was observed between lake water level and both dissolved oxygen concentration (DO) and dissolved oxygen saturation (DO sat) in Lake Markakol (*r* = −0.92, *ρ* = −0.90, *p* ≤ 0.05).

A strong negative correlation was observed between dissolved carbon dioxide (CO_2_) and phosphate (PO_4_^3−^) concentrations according to Spearman’s rank correlation coefficient (ρ = −0.96, *p* = 0.01, n = 5). A strong positive correlation was detected between ammonium (NH_4_^+^) and nitrite (NO_2_^−^) concentrations based on Pearson’s correlation coefficient (r = 0.94, *p* = 0.01, n = 5).

In addition, a strong negative relationship was found between water level and dissolved oxygen concentration (DO) (r = −0.92, *p* = 0.02; ρ = −0.90, *p* = 0.03, n = 5). A similar negative association was observed between water level and dissolved oxygen saturation (DO sat), although statistical significance was confirmed only by Spearman’s correlation analysis (ρ = −0.90, *p* = 0.03, n = 5), whereas the Pearson correlation coefficient was not significant (r = −0.84, *p* = 0.06).

No statistically significant correlations were identified between water level and CPUE, or between water level and the number of specimens recorded in catches (*p* > 0.05).

### 3.5. Population Structure and Biological Characteristics of Markakol Lenok

#### 3.5.1. Age Composition of Lenok

**Figure 9:**
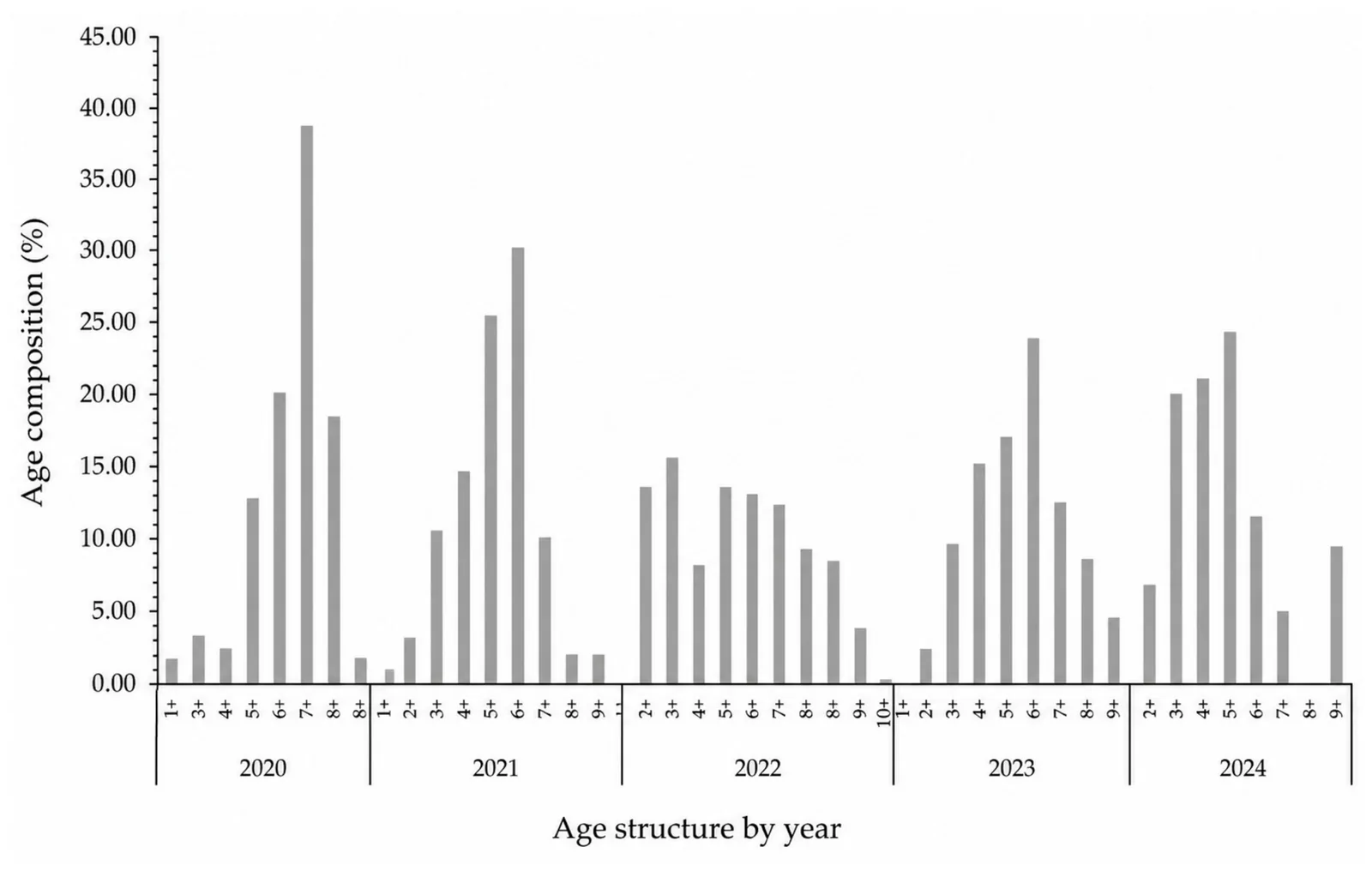
Interannual variation in the age structure of lenok in Lake Markakol during 2020–2024.

Substantial interannual variation was observed in the age structure of lenok catches, reflected in changes in the abundance and relative proportions of individual age classes and resulting in shifts in the population’s age core (Figure 9). In 2020–2021, the population was dominated by fish aged 6+ and 7+ years, whose combined proportion exceeded 55%. In 2022 and 2023, the age core was represented primarily by individuals aged 5+–7+ years, accounting for 38.57% and 50.80% of the population, respectively. A further shift towards younger age classes was observed in 2024, when fish aged 4+–6+ years became dominant, comprising more than 60% of all individuals. These findings indicate a dynamic age structure, characterised by periodic shifts in the population’s age core towards both older and younger age classes over time.

Throughout the study period, catches were dominated by individuals belonging to older age classes, whereas the proportion of lenok aged 1+–3+ years remained relatively low. Thus, the observed age structure indicates the stable presence of a broad range of age classes within the lenok population, despite interannual variation in their relative abundance. The shifts in the population’s age core, together with the predominance of fish from intermediate and older age classes in the catches, reflect the dynamic nature of population structure and the selective characteristics of the fishing gear used during sampling.

#### 3.5.2. Length–Weight Relationship

**Figure 10:**
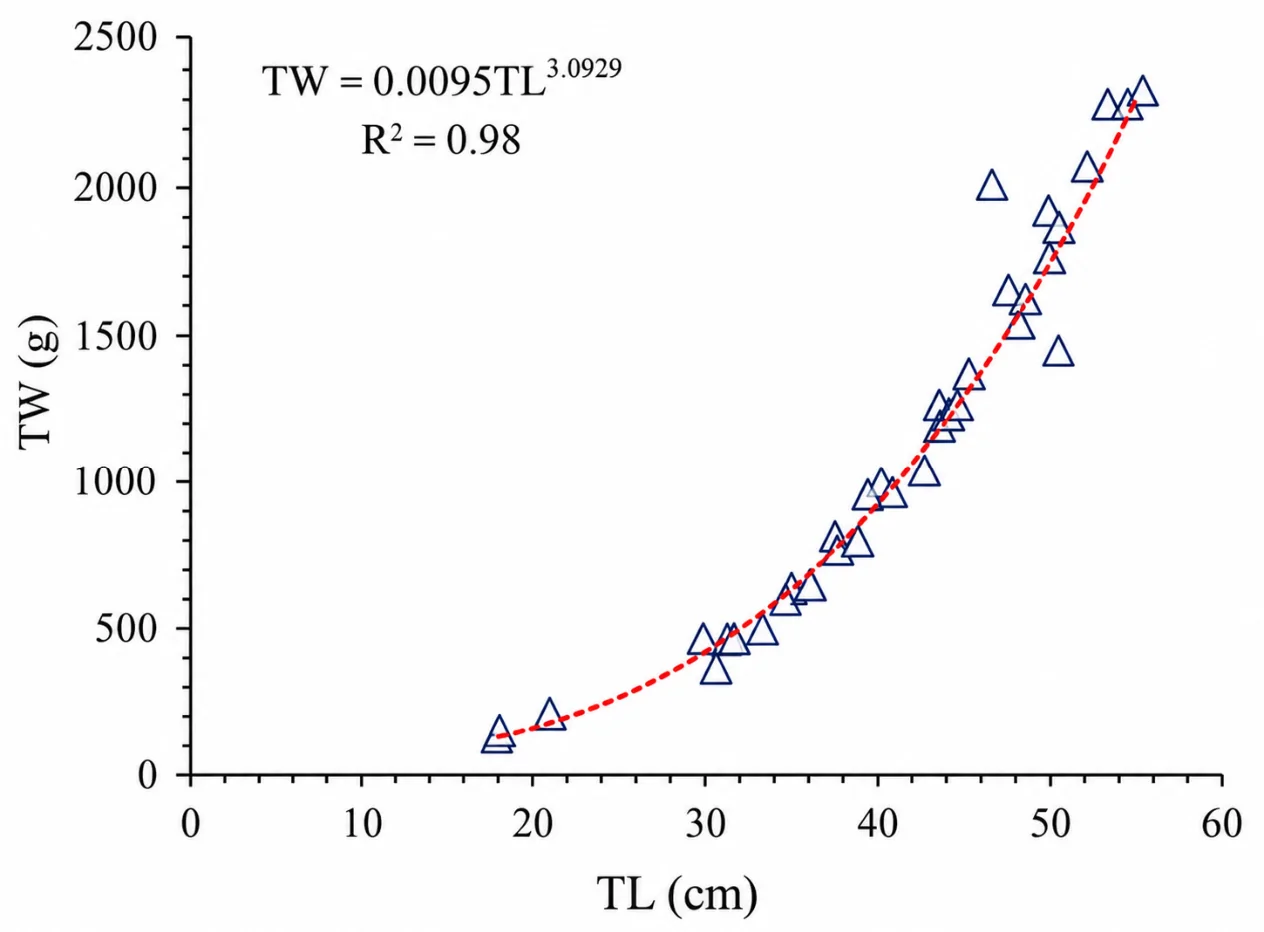
Length–weight relationship of *Brachymystax savinovi* based on mean body length and body weight values obtained from the dataset collected during 2020–2024 (N = 1993). Each point represents the corresponding mean value used in the analysis. The regression equation and R^2^ were calculated from the same mean values. A lower R^2^ would be expected if the regression were calculated using all 1,993 individual measurements.

The pooled lenok sample collected during 2020–2024 exhibited a positive allometric growth pattern (Figure 10). The estimated allometric coefficient (*b* = 3.09) was slightly higher than the value expected under isometric growth (*b* = 3), indicating positive allometry. This relationship suggests that body weight increases at a slightly faster rate than body length as fish grow, resulting in larger individuals exhibiting relatively greater robustness and body condition than would be expected under isometric growth.

The high coefficient of determination (*R*^2^ = 0.98) indicates a strong and consistent length–weight relationship within the studied population and supports the use of the derived equation for estimating body weight from body length in Markakol lenok.

**Figure 11:**
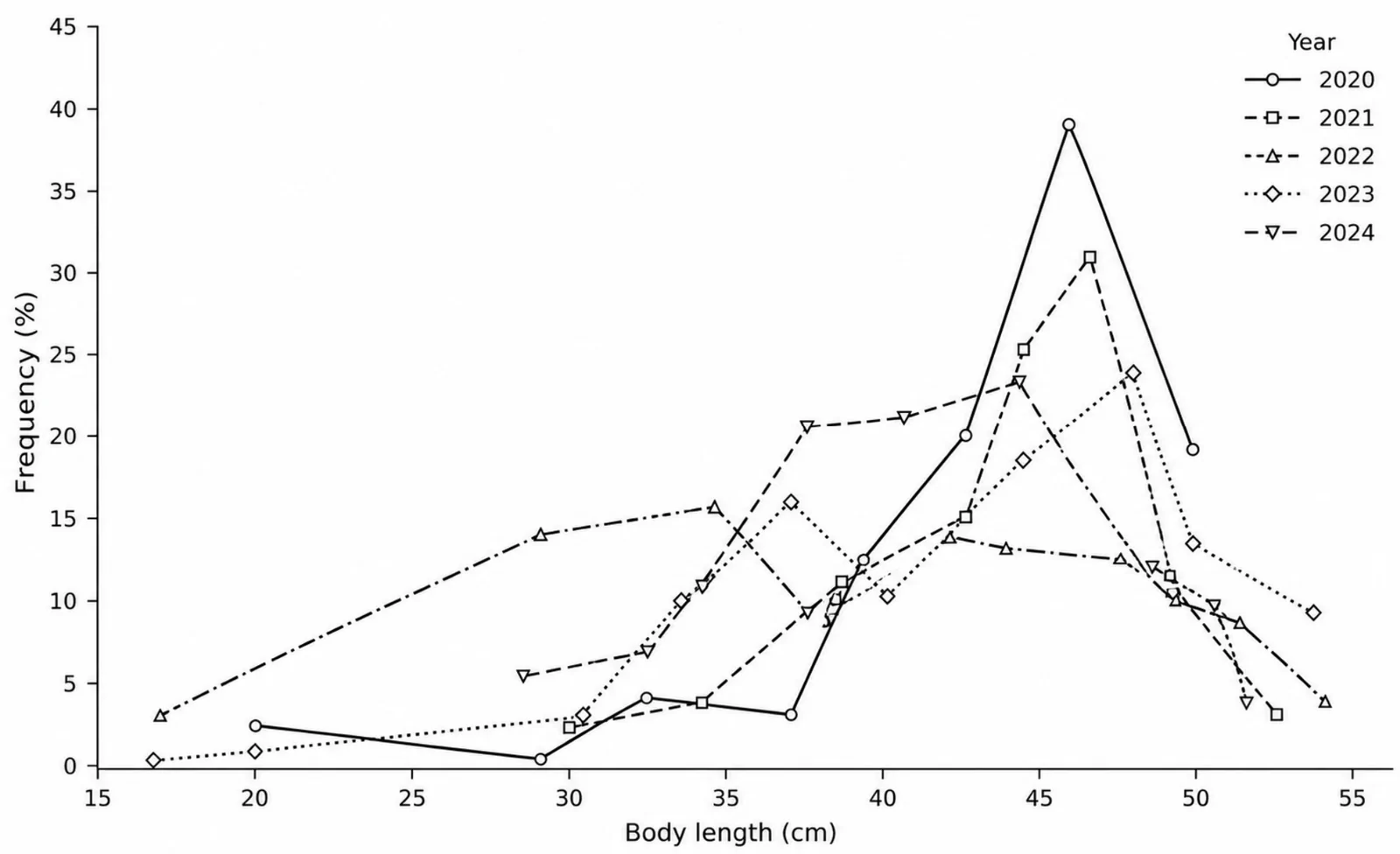
Annual frequency distribution of body length (%) in *Brachymystax savinovi* during 2020–2024.

The body length frequency distribution of *Brachymystax savinovi* varied among the study years (Figure 11). In 2020, 2021, 2023, and 2024, the distributions were generally characterised by a higher proportion of larger individuals, with the highest frequencies concentrated around 46–49 cm. In contrast, the 2022 sample showed a shift towards smaller body lengths, with the highest frequency occurring at approximately 36 cm. 

Overall, the observed interannual variation indicates differences in the size structure of the sampled population, while the predominance of larger individuals in most years suggests a relatively stable contribution of larger size classes to the annual samples.

**Figure 12:**
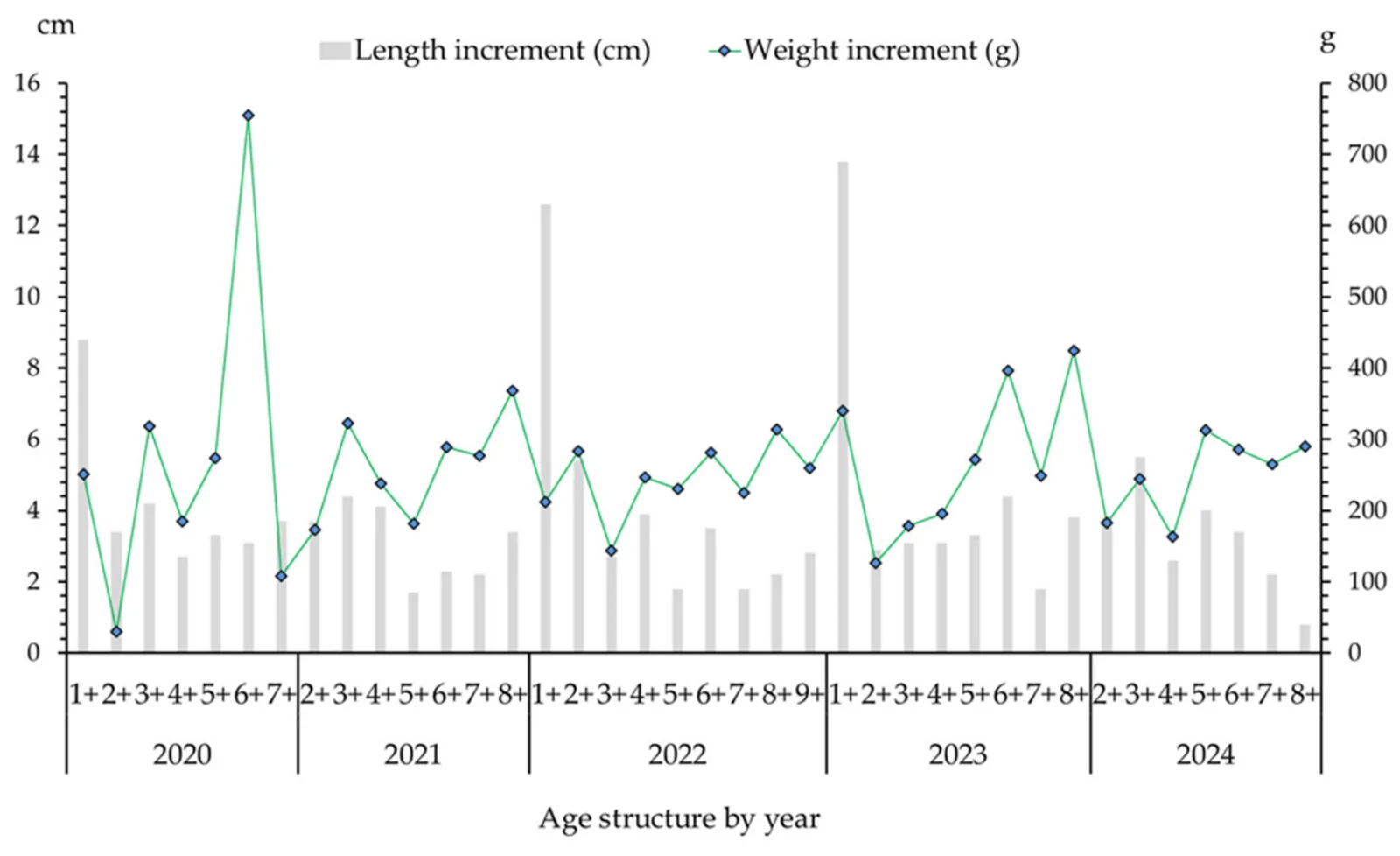
Interannual variation in age-related growth of body length and weight of lenok in Lake Markakol in 2020–2024.

Figure 12 illustrates age-related patterns of linear and weight growth in lenok, highlighting changes in the absolute increments of body length and body weight across different stages of ontogenetic development.

The most intensive linear growth of lenok was observed in the youngest age classes (1+–2+ years). The maximum absolute increment in body length was recorded for age-1+ individuals in 2023 and reached 13.80 cm, whereas the minimum value was observed in 2020 at 8.80 cm. These results indicate rapid linear growth during the early stages of development and demonstrate interannual variability in growth rates.

A marked increase in body weight was observed in Markakol lenok from age 2+ onwards, when biomass accumulation became increasingly important alongside continued linear growth. The results indicate substantially higher rates of weight gain in intermediate and older age classes, associated with the accumulation of muscle tissue, energy reserves, and the attainment of sexual maturity. This pattern reflects the characteristic shift in growth allocation observed in salmonids, from prioritising body length increase during early ontogeny to intensive body mass accumulation in later life stages.

#### 3.5.3. Fulton’s Condition Factor (FCF)

Fulton’s condition factor (FCF) in the studied lenok population ranged from 1.30 ± 0.12 in 2023 to 1.44 ± 0.26 in 2020. Variability in condition factor values was relatively low, with coefficients of variation remaining below 33% in all years examined (Table 8).

According to Fulton’s condition factor, feeding conditions for Markakol lenok can be considered favourable. Nevertheless, a gradual decline in condition factor values over time was observed, accompanied by significant interannual differences. Pairwise comparisons of mean condition factor values using Student’s *t*-test revealed statistically significant differences between most years examined (*p* < 0.05). The only exceptions were the comparisons between 2020 and 2021 (*t* = 1.91; *p* = 0.057) and between 2022 and 2023 (*t* = 1.16; *p* = 0.246), for which no significant differences were detected. The greatest difference was observed between 2021 and 2023 (*t* = 14.29; *p* < 0.001).

#### 3.5.4. Length at Maturity (L_25_, L_50_, L_75_)

**Figure 13:**
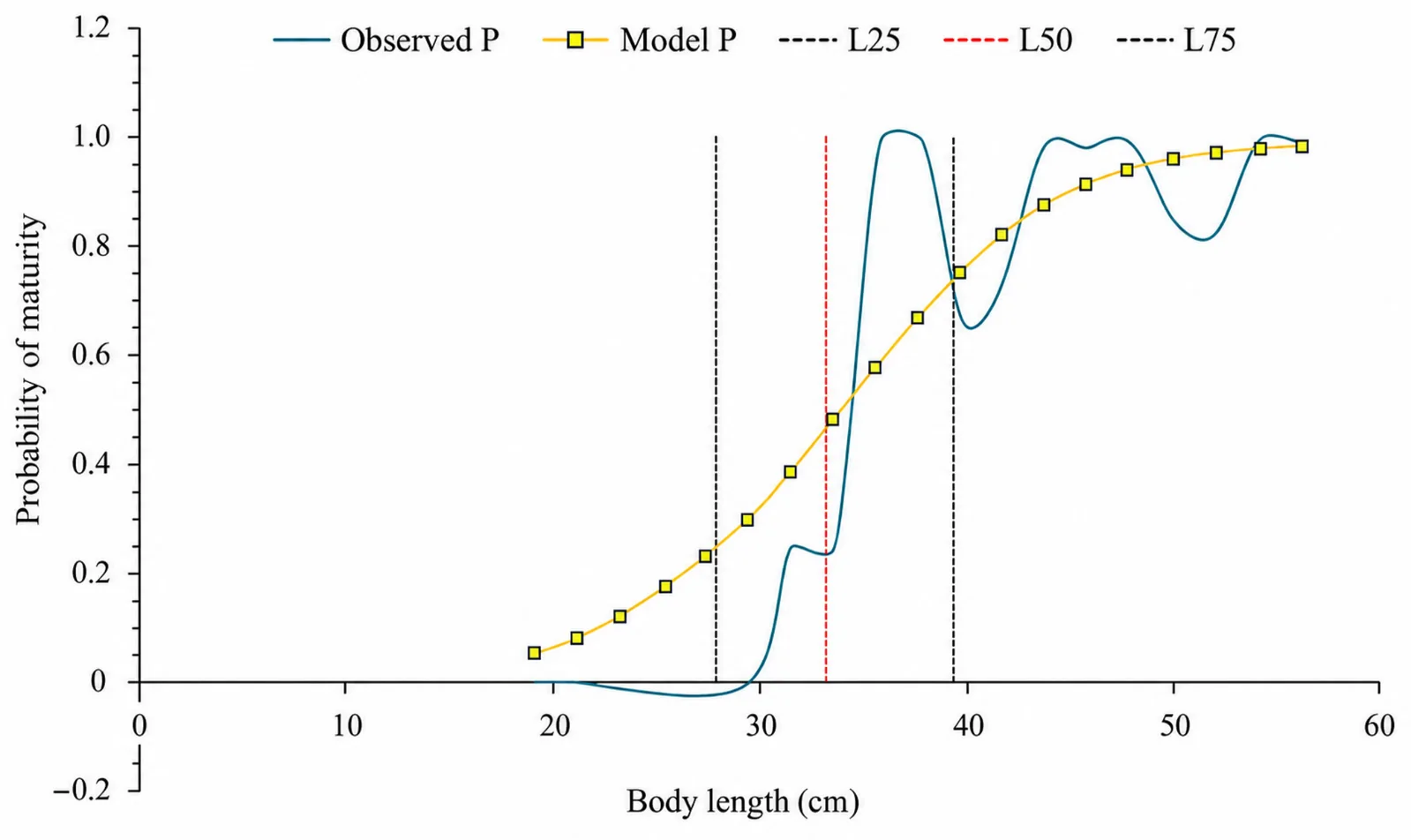
Length at maturity of lenok in Lake Markakol in 2020–2024. Observed probability of maturity (Observed P), modeled probability of maturity (Model P), and estimated lengths at 25%, 50%, and 75% maturity (L25, L50, and L75) are presented.

Using data collected during 2020–2024, size-at-maturity parameters of Markakol lenok were estimated based on body length at sexual maturity indices (L_25_–L_75_). According to the fitted logistic model (Model P), 25% of individuals attained sexual maturity at a body length of 27.10 cm (L_25_). The estimated L50 value was 33.00 cm, indicating the length at which 50% of the population reached sexual maturity. At a body length of 38.80 cm (L_75_), the proportion of mature individuals increased to 75%. Figure 13 illustrates the size distribution of lenok during the transition to sexual maturity.

The estimated size-at-maturity parameters can be interpreted in relation to the age structure of the Markakol lenok population. According to the fitted model, the onset of sexual maturity occurs at a body length of 27.10 cm (L_25_), corresponding approximately to individuals aged 4+–5+ years. The L_50_ value of 33.0 cm represents the length at which 50% of the population has attained sexual maturity and is predominantly associated with fish aged 6+ years and older. These findings indicate that the majority of Markakol lenok enter the reproductive phase from the age of 6+ years onwards, whereas the first mature individuals may occur as early as the 4+–5+ age classes.

It should be noted that individuals belonging to these age classes formed the core of the Markakol lenok population during 2020–2024 and accounted for the majority of fish captured, particularly those aged 5+–8+ years. These age classes make the greatest contribution to population recruitment and persistence, as they comprise a substantial proportion of sexually mature individuals. Consequently, prolonged fishing pressure may lead to changes in the age and size structure of the population, as well as shifts in maturation parameters, including reductions in the mean age and body length at sexual maturity. Such changes may have important implications for the reproductive potential and long-term stability of the population.

### 3.6. Formation of the Reproductive Stock of Lenok

To support population restoration of Markakol lenok, a series of measures was undertaken to establish a replacement and broodstock population (RBP) using fish originating from the Uidene River Reservoir. Rearing conditions are described in the Section 2. Based on the results of broodstock assessment conducted in 2022, two groups of lenok were formed and transferred to two aquaculture facilities. The results of the biological analyses are presented in Table 9 and Table 10.

At the beginning of the experiment, the mean body weight of lenok reared at OstFish LLP was 503.0 g. During the 75-day rearing period, a consistent increase in body weight was observed. The absolute weight gain reached 123.0 g, indicating favourable rearing conditions and efficient feed utilisation.

No mortality was recorded throughout the rearing period, corresponding to a survival rate of 100% for the Uidene lenok stock. The absence of mortality indicates satisfactory physiological condition of the fish, favourable hydrochemical conditions, and the suitability of the rearing environment for the biological requirements of the species.

The second group, maintained at Grand-FISH LLP, was characterised by smaller body size and weight compared with the first group. By the end of the rearing period, the mean body weight had reached 335.17 ± 74.50 g. No mortality was recorded throughout the experiment. Adaptation to artificial feeds enabled the fish to maintain a stable growth rate over the 120-day rearing period, achieving an average daily weight gain of 0.90 ± 0.15 g day^−1^.

The conditions provided in the flow-through rearing tanks ensured 100% survival of the Uidene lenok and facilitated their successful adaptation to artificial feeds.

The biological data obtained from lenok originating from the Uidene River Reservoir were subsequently used for the establishment of a replacement and broodstock population and for the selection of brood fish for artificial propagation. Subsequent breeding operations resulted in the production of viable offspring under controlled conditions, confirming the reproductive suitability of the selected broodstock. Detailed information on egg incubation conditions and juvenile production performance has been reported in studies conducted by researchers of the Altai Branch of the Fisheries Research and Production Centre LLP [45].

## 4. Discussion

At present, in addition to natural environmental factors, the abundance of Markakol lenok is influenced by fishing pressure. According to published data, the size of the spawning stock at the beginning of the 2000s was approximately 1.5 times lower than that recorded during the 1980s and 1990s [1,46]. Previous studies and observations from Lake Markakol have linked this decline to both legal and illegal fishing activities, as well as to violations of the protected-area management regime and insufficient enforcement of conservation measures [47,48,49]. These factors are considered important drivers affecting the long-term status and sustainability of the population.

According to published sources, commercial fisheries targeting lenok and grayling were conducted in Lake Markakol during the early 1980s, including periods coinciding with spawning migrations [50]. Total catches reached approximately 300 t in 1982 and remained substantial in subsequent years. Fishing activities were concentrated in the mouths of major spawning tributaries, potentially limiting access of spawners to breeding habitats. Such fishing pressure has been identified as an important factor affecting the abundance and age structure of lenok and grayling populations in Lake Markakol [14].

At present, lenok fishing in Lake Markakol is permitted only for local residents and is regulated under the specific conservation requirements applicable within the protected area [51]. These management measures help to limit fishing pressure on the lenok population and contribute to maintaining its structural integrity. Nevertheless, the long-term monitoring results presented in this study indicate ongoing changes in the age and size structure of the population.

Analysis of age composition showed that the reproductive core of the population is primarily formed by individuals aged 5+–8+ years, which contribute most substantially to population recruitment. At the same time, the proportion of older fish (9+ years and older) remains extremely low, suggesting increased vulnerability of the population to the removal of large spawning individuals. A decline in the abundance of these fish may reduce spawning stock size and decrease the resilience of the population to external pressures. According to previous studies, the proportions of lenok aged 9+ and 10+ years were only 1.5% and 0.3%, respectively, corresponding to the occurrence of merely one or two individuals in the sampled catches [5]. Such an age–size structure may be considered an indicator of the selective removal of larger fish and highlights the need for continued monitoring of the population, particularly under conditions of ongoing anthropogenic pressure. At the same time, the selectivity of the fishing gear used, especially with respect to mesh size, should be taken into account when interpreting these patterns. For example, the estimated body length at which approximately 50% of lenok attain sexual maturity (L50) was 33.0 cm. This finding emphasises the need to develop appropriate fishery regulations and to establish a minimum legal catch size for the species. Consequently, prolonged fishing pressure may lead to changes in the age and size structure of the population, as well as shifts in maturation parameters, including reductions in the mean age and body length at sexual maturity. Such changes may have important implications for the reproductive potential and long-term stability of the population.

When considered together with seasonal patterns in catch per unit effort (CPUE) and the aggregated distribution of lenok throughout Lake Markakol (AI ≥ 1), additional risks of localised overexploitation may arise. Seasonal variation in the distribution of lenok may be associated with differences in water depth across the lake. It is known that the Matabai and Elovka stations are characterised by relatively deeper habitats [48]. However, it was not possible to evaluate the relationship between seasonal lenok distribution and the bathymetry of Lake Markakol owing to the lack of appropriate depth-related data.

Environmental conditions influencing the status of the lenok population appear to be generally favourable for both survival and reproduction when compared with previously available data. For example, the water level of Lake Markakol in 2024 was higher than in preceding years and, according to available information, this increase was associated with intensive snowmelt and enhanced inflow of meltwater during mid-spring and early summer [13]. The increase in water level observed during 2023–2024 may have created more favourable conditions for the functioning of the aquatic ecosystem and the maintenance of biodiversity within Lake Markakol. Changes in the hydrological and hydrochemical characteristics of Lake Markakol have also been linked to rising temperatures, transboundary transport of pollutants, and the overall ecological condition of the ecosystem [14].

The increase in certain hydrochemical parameters with depth may be related to the accumulation of organic matter in bottom layers, its mineralisation, and restricted water exchange, all of which contribute to elevated concentrations of oxidisable organic compounds. The elevated PI values observed in 2022 may indicate a temporary increase in the input of organic matter into the lake ecosystem. However, the relatively low interannual variability of both PI and TDS suggests that the hydrochemical regime of Lake Markakol remained generally stable throughout the study period. Furthermore, nutrient concentrations consistently remained below the permissible thresholds established for fishery water bodies [44], indicating the absence of pronounced nutrient enrichment and supporting the overall stability of water quality conditions.

Body length and weight characteristics also support the observed growth pattern of Markakol lenok. The established length–weight relationship indicates that body mass increases proportionally faster than body length as individuals grow. Similar growth patterns have been reported for salmonid fishes, where linear growth predominates during the early stages of ontogeny, whereas biomass accumulation becomes more pronounced with increasing age and size. Such growth characteristics are generally associated with favourable feeding conditions and adequate energy allocation for somatic growth. In addition, age-related increases in body weight may reflect the utilisation of larger and more energy-rich food resources by older individuals. Together, these findings suggest that Lake Markakol continues to provide suitable trophic conditions for the growth and development of Markakol lenok.

A synthesis of published information and the results of the present study indicates that the lenok population in Lake Markakol has undergone substantial changes in age structure as a consequence of prolonged intensive fishing pressure. At present, the population may be regarded as relatively stable, a condition that is likely associated with existing management measures and the regulation of harvest levels through established catch quotas. Nevertheless, current abundance estimates and the principal biological characteristics of lenok suggest that favourable conditions remain for the conservation and further recovery of the population.

In this context, the establishment of replacement and broodstock populations, including those based on fish originating from the Uidene River Reservoir, is of particular importance. Such initiatives are aimed at ensuring the sustainable production of viable stocking material for artificial propagation and restocking programmes. Measures designed to restore stocks of valuable commercial fish species, as well as endemic, rare, and threatened taxa, constitute an important component of contemporary biodiversity conservation strategies and the sustainable management of aquatic biological resources [51,52,53,54].

Within the framework of the present study, biological characteristics of lenok originating from the Uidene River Reservoir and subsequently adapted to industrial aquaculture conditions at fish farms in Kazakhstan were evaluated. The results demonstrated a consistent positive growth response, indicating successful domestication and a high capacity of the species to adapt to artificial rearing conditions. These findings confirm the feasibility of establishing replacement and broodstock populations of lenok and their subsequent use in artificial propagation and population restoration programmes.

Given the long-term isolation and unique biological characteristics of Markakol lenok, the conservation of its natural population is of both ecological and practical importance. This population represents a valuable source for the development of replacement and broodstock populations, the implementation of artificial propagation programmes, and the preservation of the subspecies’ genetic diversity. Furthermore, it may contribute to the advancement of scientifically based approaches to cold-water aquaculture in the region [55].

A promising direction for future research is the evaluation of the effectiveness of artificial propagation and restocking of Lake Markakol with juvenile lenok, followed by long-term monitoring of their survival, growth, and contribution to the replenishment of the natural population. Such data would enable an objective assessment of the effectiveness of population restoration measures implemented for this species.

At the same time, several aspects of lenok artificial propagation technology remain insufficiently developed. In particular, there is currently no scientifically validated regulatory and technological framework governing the establishment of replacement and broodstock populations, the collection of gametes, egg incubation procedures, and juvenile rearing practices. The development of such a framework represents an important prerequisite for improving the effectiveness of programmes aimed at the conservation and restoration of natural lenok populations.

Although historical records indicate that the lenok population in the Uidene River Reservoir was established from juveniles originating from Lake Markakol, the genetic correspondence between the current broodstock population from the Uidene River Reservoir and the original Markakol lenok population has not yet been confirmed using molecular genetic methods. Therefore, at this stage, it cannot be conclusively established that the Uidene River Reservoir broodstock fully retains the genetic characteristics of the original Lake Markakol population. Accordingly, one of the priorities for future research should be to conduct molecular genetic analyses using appropriate diagnostic genetic markers to assess the degree of genetic similarity and verify the origin of the broodstock population. Such analyses will provide an important scientific basis for the informed use of this broodstock in artificial propagation, conservation, and restoration programmes for the Markakol lenok population.

One of the promising approaches for the conservation of *Brachymystax savinovi* is the supplementation of the natural population with juveniles produced under aquaculture conditions from a reproducing broodstock established using breeders originally collected from Lake Markakol.”

## 5. Conclusions

1. The hydrochemical characteristics of Lake Markakol remained stable during 2020–2024 and corresponded to those of an oligotrophic cold-water lake. Nutrient concentrations, permanganate index values, and total dissolved solids indicated favourable water quality and the absence of significant anthropogenic impact, confirming the overall ecological stability of the lake.
2. The hydrological regime of Lake Markakol was characterized by relatively stable water levels, with fluctuations remaining within the range of natural interannual variability. The temporary decline observed in 2022 was followed by recovery during 2023–2024, indicating the maintenance of a stable water balance and favourable conditions for ecosystem functioning.
3. The contemporary population of Markakol lenok was dominated by intermediate age classes (5+–8+ years), whereas older individuals were comparatively scarce. Despite this shift, positive allometric growth, satisfactory condition factors, and a considerable proportion of mature fish indicate that the population remains relatively stable and retains substantial reproductive potential. However, for the long-term sustainability of the population, continued monitoring of age structure, appropriate regulation of fishing pressure, and further development of artificial propagation programmes should be considered.
4. Lenok originating from the Uidene River Reservoir successfully adapted to industrial aquaculture conditions, achieving 100% survival in both rearing systems. The higher growth rates observed in recirculating aquaculture systems demonstrate their potential for broodstock development and artificial propagation, supporting the conservation and sustainable management of the species.

## Figures and Tables

**Table 1:** Geographic Coordinates and General Characteristics of Lenok Sampling Stations in Lake Markakol during 2020–2024.

Station Name	Coordinates	Lake Sector	Approximate Depth (m)	Bottom Substrate	Distance From Shore (m)	Habitat Characteristics
Urunkhaika	48°47′7.94″ N; 86°1′4.46″ E	Eastern shore	3–10	Gravelly–rocky	100–500	Steeper nearshore profile, influenced by river discharge
Topolevka	48°48′9.17″ N; 85°56′17.21″ E	Northeastern shore	2–5	Sandy–gravelly with silt deposits	50–200	River mouth zone, gently sloping littoral area
Yelovka	48°48′6.99″ N; 85°39′56.31″ E	Eastern shore	2–6	Sandy–gravelly	50–250	Shallow littoral habitat influenced by river inflow
Matabay	48°40′23.65″ N; 85°39′5.98″ E	Southern shore	3–7	Gravelly–rocky with sandy patches	100–300	Open coastal area with moderate wave exposure

**Table 2:** Mean Hydrochemical Characteristics of Lake Markakol over the Period 2020–2024.

Year	pH	CO_2_ (mg/L)	DO (mg/L)	DO sat (%)	NH_4_^+^ (mg/L)	NO_2_^−^ (mg/L)	NO_3_^−^ (mg/L)	PO_4_^3−^ (mg/L)	PI (mg O/L)	TDS (mg/L)
2024	8.40 ± 0.08	0.10 ± 0.02	8.34 ± 0.25	85.50 ± 3.80	<0.20	0.02 ± 0.00	0.22 ± 0.02	0.02 ± 0.00	1.74 ± 0.14	38.00 ± 1.50
2023	7.50 ± 0.07	0.10 ± 0.01	8.30 ± 0.28	86.00 ± 4.00	0.20 ± 0.03	0.01 ± 0.00	0.27 ± 0.02	0.02 ± 0.00	1.54 ± 0.12	33.00 ± 1.70
2022	7.80 ± 0.09	0.10 ± 0.01	9.39 ± 0.35	94.50 ± 4.50	0.26 ± 0.04	0.02 ± 0.00	0.26 ± 0.03	0.02 ± 0.00	1.93 ± 0.18	63.00 ± 2.80
2021	7.70 ± 0.08	0.10 ± 0.02	8.79 ± 0.32	93.30 ± 4.20	0.25 ± 0.04	0.02 ± 0.00	0.66 ± 0.05	0.05 ± 0.01	1.58 ± 0.13	46.00 ± 2.00
2020	7.50 ± 0.07	0.10 ± 0.03	8.73 ± 0.30	93.50 ± 4.30	0.54 ± 0.08	<0.10	<0.01	0.07 ± 0.01	1.65 ± 0.15	57.00 ± 2.50

**Table 3:** Linear Regression Analysis of Interannual Trends in Hydrochemical Parameters of Lake Markakol (2020–2024).

Parameter	Slope (B)	R^2^	*p*-Value	Trend
pH	−1.60	0.46	0.203	Negative (weak)
DO	1.64	0.20	0.440	Positive (weak)
DO sat.	0.29	0.69	0.079	Positive (moderate)
NH_4_^+^	0.81	0.65	0.096	Positive (moderate)
NO_2_^−^	0.72	0.52	0.169	Positive (weak)
NO_3_^−^	0.53	0.29	0.348	Positive (weak)
PO_4_^3−^	0.89	0.79	0.410	Positive (moderate)
PI	−1.45	0.02	0.819	No trend
TDS	0.64	0.41	0.244	Positive (weak)

Note. Values are presented as regression slope (B), coefficient of determination (R^2^), and significance level (*p*-value). Statistical significance was set at *p*< 0.05.

**Table 4:** Linear regression analysis of water levels in Lake Markakol during 2020–2024.

Year	Water Level, m	CV (%)	Slope (B)	R^2^	*p*-Value	Trend
	Mean ± SD					
2020	47.63 ± 0.07	0.15	−0.20	0.04	0.52	Slight decreasing trend (ns)
2021	47.53 ± 0.10	0.20	−0.50	0.25	0.09	Moderate decreasing trend (ns)
2022	47.45 ± 0.10	0.22	−0.36	0.13	0.24	Weak negative trend (ns)
2023	47.67 ± 0.12	0.22	0.67	0.46	0.01	Significant increasing trend
2024 (9 month)	47.74 ± 0.18	0.25	−0.25	0.07	0.50	Slight decreasing trend (ns)

Note. Values are presented as the regression slope (B), coefficient of determination (R^2^), significance level (*p*-value), ns—not significant. Statistical significance was accepted at *p* ≤ 0.05.

**Table 5:** Relationship between the abundance and biomass of fish catches in Lake Markakol during 2020–2024.

Station Name	Total Catch Weight (kg)	Cath Count (ind.)	r (Pearson)	r (Strength of Association)
	M ± SD			
URK	25.70 ± 18.01	21.61 ± 12.92	0.93	Strong positive correlation
ELO	20.55 ± 8.20	22.32 ± 8.97	0.77	Strong positive correlation
TOP	27.58 ± 24.74	24.61 ± 16.15	0.90	Strong positive correlation
MAT	23.84 ± 10.14	22.48 ± 7.58	0.83	Strong positive correlation

Note. Pearson correlation coefficients were significant at the 0.01 probability level (*p* < 0.01). URK—Urunkhaika, TOP—Topolevka, MAT—Matabai, ELO—Yelovka.

**Table 6:** Aggregation index of *Brachymystax savinovi* catches in Lake Markakol during 2020–2024.

Year	Season	CV (%)	Aggregation Index (AI)
2020	Spring	29.06 ± 13.85	5.21 ± 2.22
2021	Summer	39.54 ± 15.15	3.84 ± 2.37
2022	Spring	24.53 ± 15.04	1.52 ± 2.35
2022	Autumn	18.26 ± 14.72	1.31 ± 2.31
2023	Summer	40.35 ± 14.68	3.14 ± 2.27
2023	Autumn	33.48 ± 14.60	1.62 ± 2.23
2024	Summer	30.09 ± 14.38	1.49 ± 2.19
2024	Autumn	21.84 ± 14.11	0.88 ± 2.40

**Table 7:** Correlations between hydrological and hydrochemical parameters and Markakol lenok abundance and CPUE in 2020–2024.

Comparisons	Pearson r	*p*-Value	Spearman ρ	*p*-Value	N
Water level–CPUE	0.03	0.92	0.03	0.93	9
Water level-specimens	−0.28	0.45	−0.43	0.24	9
Water level-DO	−0.92	0.02	−0.90	0.03	5
Water level–DO sat	−0.84	0.06	−0.90	0.03	5
CO_2_-PO_4_^3−^	0.08	0.89	−0.96	0.01	5
NH_4_^+^-NO_2_^−^	0.94	0.01	0.80	0.10	5

Note. Statistical significance for all comparisons was set at *p* ≤ 0.05.

**Table 8:** Mean condition factor values of lenok (*Brachymystax savinovi*) from Lake Markakol in different years.

Year	Mean ± SD	CV (%)	n
2020	1.44 ± 0.26	17.88	308
2021	1.41 ± 0.09	6.25	306
2022	1.31 ± 0.13	9.83	407
2023	1.30 ± 0.12	9.10	439
2024	1.38 ± 0.07	5.03	336

**Table 9:** Biological performance indicators of lenok reared in recirculating aquaculture systems (RAS) from September to December 2022 at the «OstFish» LLP facility.

Parameters	Min	Max	Mean ± SD	N
Initial weight, g	503.00	1122.00	874.12 ± 153.20	47
Final weight, g	575.00	1245.00	916.17 ± 164.32	
Absolute weight gain, g	72.00	123.00	93.28 ± 13.15	
Average daily weight gain, g/day	1.20	2.00	1.60 ± 0.20	

**Table 10:** Biological performance indicators of lenok reared in pools from September to December 2022 at the «GrandFish» LLP facility.

Parameters	Min	Max	Mean ± SD	N
Initial weight, g	138.00	417.00	283.13 ± 69.75	58
Final weight, g	181.00	479.00	335.17 ± 74.50	
Absolute weight gain, g	43.00	62.00	52.05 ± 4.75	
Average daily weight gain, g/day	0.70	1.30	0.90 ± 0.15	

## Data Availability

The original contributions presented in this study are included in the article. Further inquiries can be directed to the corresponding author.

## Abbreviations

The following abbreviations are used in this manuscript:

RK	Republic of Kazakhstan
IUU	Illegal, Unreported and Unregulated (Fishing)
CPUE	Catch Per Unit Effort
FCF	Fulton Condition Factor
